# Application of filamentous phages in environment: A tectonic shift in the science and practice of ecorestoration

**DOI:** 10.1002/ece3.4743

**Published:** 2019-01-25

**Authors:** Radhey Shyam Sharma, Swagata Karmakar, Pankaj Kumar, Vandana Mishra

**Affiliations:** ^1^ Bioresources and Environmental Biotechnology Laboratory, Department of Environmental Studies University of Delhi Delhi India

**Keywords:** bioremediation, biosensors, ecological theory, filamentous phages, microbial ecology and fitness, restoration ecology

## Abstract

Theories in soil biology, such as plant–microbe interactions and microbial cooperation and antagonism, have guided the practice of ecological restoration (ecorestoration). Below‐ground biodiversity (bacteria, fungi, invertebrates, etc.) influences the development of above‐ground biodiversity (vegetation structure). The role of rhizosphere bacteria in plant growth has been largely investigated but the role of phages (bacterial viruses) has received a little attention. Below the ground, phages govern the ecology and evolution of microbial communities by affecting genetic diversity, host fitness, population dynamics, community composition, and nutrient cycling. However, few restoration efforts take into account the interactions between bacteria and phages. Unlike other phages, filamentous phages are highly specific, nonlethal, and influence host fitness in several ways, which make them useful as target bacterial inocula. Also, the ease with which filamentous phages can be genetically manipulated to express a desired peptide to track and control pathogens and contaminants makes them useful in biosensing. Based on ecology and biology of filamentous phages, we developed a hypothesis on the application of phages in environment to derive benefits at different levels of biological organization ranging from individual bacteria to ecosystem for ecorestoration. We examined the potential applications of filamentous phages in improving bacterial inocula to restore vegetation and to monitor changes in habitat during ecorestoration and, based on our results, recommend a reorientation of the existing framework of using microbial inocula for such restoration and monitoring. Because bacterial inocula and biomonitoring tools based on filamentous phages are likely to prove useful in developing cost‐effective methods of restoring vegetation, we propose that filamentous phages be incorporated into nature‐based restoration efforts and that the tripartite relationship between phages, bacteria, and plants be explored further. Possible impacts of filamentous phages on native microflora are discussed and future areas of research are suggested to preclude any potential risks associated with such an approach.

## 1 INTRODUCTION

Theories in soil biology guide the efforts to restore vegetation in degraded habitats. Natural attenuation—banning any activity that results in environmental degradation—is useful only in those ecosystems that are in the early stages of degradation. Currently, a vast majority of degraded ecosystems show altered abiotic and biotic components and lowered resilience. Consequently, assisted restoration practices, such as planting native species, replacing or treating contaminated soil, and managing water resources, represent the only land restoration options for most of the degraded ecosystems. The Society of Ecological Restoration defines ecorestoration as “*the process of assisting the recovery of an ecosystem that has been degraded, damaged or destroyed*” (SER, 2004). Restoration practices developed on the principles of plant–microbe mutualism and microbial cooperation, synergism, and antagonism have facilitated ecorestoration (Heneghan et al., 2008; Perring et al., 2015; Young, Petersen, & Clary, 2005).

Over 65% of the earth's surface has been degraded or contaminated, which has resulted in the loss of its potential to benefit human society—which is why ecorestoration is held to be a global need by the United Nations. Restoration practices have site‐specific goals, such as to repair environmental damage after deforestation or overharvesting, to stabilize soil after mining, to restore the productivity of saline soils after heavy irrigation, and to remediate soils contaminated as a result of industrial activity. However, major goals of all land restoration continue to be the restoration of biodiversity, revival of ecosystem services for socio‐economic security, and improved resilience of ecosystems to future environmental change. Restoration may be voluntary, undertaken to improve the quality of life, or mandatory, a legislative directive to ensure sustainable development.

**Figure 1:**
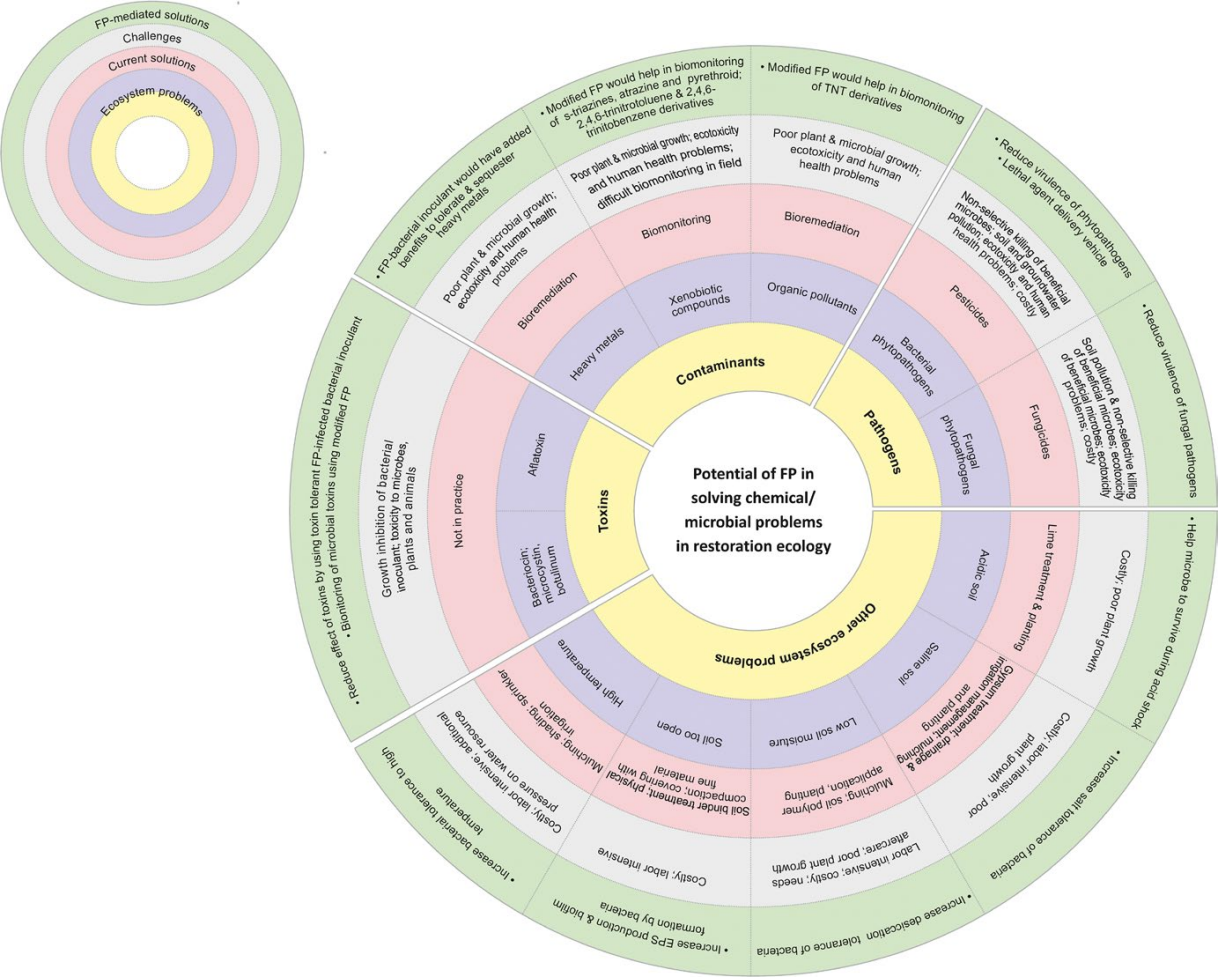
Ecosystem and other environmental challenges for ecological restoration of degraded lands, where current techniques face challenges but filamentous phage has potential to provide solutions. FP, filamentous phage

Degraded lands need specialized restoration efforts rather than conventional soil amelioration methods, because such lands are often exposed to multiple sources of stress such as high levels of contaminants, toxins, and pathogens (Perring et al., 2015). Traditionally, soil scientists use agronomic practices and chemical amendments to improve soil fertility (Filiberto & Gaunt, 2013), although different sources of stress need source‐specific efforts to improve soil and plant health. The traditional methods commonly used for treating farmlands or small patches of degraded lands are not only too costly for restoring vast stretches of degraded ecosystems but also of limited efficacy in controlling pathogens and biological toxins and coping with changes in the environment (Figure 1). In contrast, microbial inocula (free‐living, associative, and endosymbiotic) repair normal biological processes affected by degradation, ameliorate contaminated soils, and control phytopathogens in degraded habitats. Microbial inocula are thus an ecologically sound option to speed up revegetation and revive ecosystem services under diverse environmental regimes (Tables 1, 2, 3). Some bacterial genera, such as *Bacillus*, *Bradyrhizobium*, *Enterobacter*, and *Pseudomonas*, have been widely used as inocula and even commercial formulations have been developed for use with economically and ecologically important plants (Table 1). Microbes may possess multiple traits that may be deployed in combating both abiotic and biotic sources of stress; however, restoration ecologists select bacteria with specific traits to tackle the most serious environmental challenges (Rau et al., 2009; Sharma, Mishra, Rau, & Sharma, 2015). Alternatively, consortia of microbes may also be developed to deal with multiple challenges, but their efficacy is reduced because members of such consortia differ in their environmental and nutritional requirements, and the use of consortia continues to face unpredictable challenges.

**Table 1:** Potential bacterial targets to explore filamentous phage to improve the benefits of commercial inoculants being used to promote plant growth under different environmental stresses

Target function	Target organism(s)/activity	Bacterial composition	Trade name of inoculants	Filamentous phage
Biofungicide	Rot diseases caused by *Fusarium* and *Rhizoctonia*	*Bacillus subtilis*	Kodiak HB	Needs investigation
	Seed‐borne disease caused by fungus *Ascochyta* spp.	*Pseudomonas chlororaphis*	Cedress; Cedomon	Known for *Pseudomonas*
	Rots, blights, wilt, leaf spot, and mildew	*Bacillus subtilis*	Biotilis	Needs investigation
	Leaf spot caused by different genera of fungus	*Pseudomonas fluorescens*	Bactvipe	Known for *Pseudomonas*
	Seed‐borne diseases: Common wheat bunt (*Tilletia caries*), wheat leaf spot (*Septoria nodorum*)	*Pseudomonas chlororaphis*	Cerall	Known for *Pseudomonas*
Bioinsecticide	Lepidoptera larvae and beetles	*Bacillus thuringiensis*var. *kurstaki*	Bioscrop BT16	Needs investigation
	Insecticide/miticide: Infection by aphids, psyllids, whiteflies, lygus and mealybugs, thrips and phytophagous mites	*Chromobacterium subtsugae strain*PRAA4‐1	Grandevo	Needs investigation
	Insect pests of lepidoptera, diptera, coleoptera, hymenoptera	*B. thuringiensis var. kurstaki*	Lipel	Needs investigation
Bionematicides	*Meloidogyne* spp, *Hetrodera* spp, *Helicotylenchus* spp, *Hoplolaimus* spp. causing root‐knot, cyst, lesion	*Bacillus firmus*	Bionemagon	Needs investigation
Multifunctional PGPR	Fire blight (*Erwinia amylovora*) Antibiosis (via production of siderophore) Biocontrol of fire blight Protection from frost damage	*Pseudomonas fluorescens*A506	BlightBan A506	Known for *Pseudomonas*
	Biofungicide (sheath blight caused by several fungal species) Bionematicide (root‐knot, cyst) Biocontrol of bacterial pathogens (via production of siderophores and hydrogen cyanide)	*P. fluorescens*IIHR PF‐2	Sheathguard	Known for *Pseudomonas*
	Biofungicide, N_2_‐fixer IAA producer	*Pseudomonas chlororaphis*subsp*. aurantiaca*SR1	Liquid PSA	Known for *Pseudomonas*
	N_2_‐fixer Biocontrol of pathogens	*Azotobacter Chrococcum*	Bio Azo	Needs investigation
	N_2_‐fixer Bionematicide	*Azotobacter chroococcum*, *Pseudomonas fluorescens*	Bio Gold	Known for *Pseudomonas*
	Bioinsecticide (flea beetles, leaf aphids, root worms) Bionematicide (cyst, root‐knot, root‐lesion)	*Bacillus*, *Firmus*,* Clothianidin*	Poncho/Votivo	Needs investigation
	Provide silicates Develop resistance for abiotic and biotic stresses in plants	*Bacillus species*	Si Sol B	Needs investigation
Nutrient Solubilizer/Mobilizer	N_2_‐fixation	*Bradyrhizobium japonicum*	Optimize liquid Soybean; Rhizo‐Flo	Needs investigation
		*B. japonicum*(USDA 138 for USA, 532C for Canada*)*	Nodulest 10	
		*Acetobacter diazotrophicus*	Bio Aceto	
		*Bradyrhizobium japonicum, Penicillium bilaii*	TagTeam LCO	
		*Delftia acidovorans*, *Bradyrhizobium japonicum*	Bioboost	
		*Glomus intraradices*, *Rhizobium leguminosarum*bv. *viciae*	AGTIV	
		*Bacillus subtilis*	HiStick N/T	
		*Bradyrhizobium japonicum*, *Trichoderma sp*.	Graph‐Ex SA	
		*Bradyrhizobium japonicum*	Dyna‐Start; Vault SP	
	Provide inorganic and organic nutrients	*Pseudomonas azotoformans*	Amase	
	Improved bioavailability of phosphate and uptake of N and K	*Bacillus amyloliquefaciens, Trichoderma virens*	QuickRoots	
	Provides N and P	*Acidovorax facilis, Bacillus subtilis, B. licheniformis, B. megaterium, B. oleronius, B. marinus, & Rhodococcus rhodochrous*	Accomplish LM	
	Iron mobilization	*Acidithiobacillus ferrooxidan*	Fe Sol B	
	K Mobilization through production of organic acids and enzymes	*Frateuria aurantia*	K Sol B	
	N_2_‐fixation Phosphate mineralization Increase in soil urease and dehydrogenase activity	*Azotobacter vinelandii‐B*1795*, Bacillus megaterium*B1091*, Clostridium pasteurianum, Azospirillum*sp*., Bacillus subtilis, Rhodobacter*sp*., Lactobacillus*sp*., Trichoderma reesei, Saccharomyces cerevisiae, Streptomyces*sp.	Microbion UNC	Known for *Clostridium*; needs investigation for other
		*Rhizobium leguminosarum*biovar* viceae*	Nodulator XL	Needs investigation
		*Azospirillum brasilense; Rhizobium*	Nitrofix and Bioenraiz	
		*Bradyrhizobium*sp.	Vault SP	
		Consortium of *Rhizobium*	Rhizo‐Flo	
		High load of* Rhizobium*	Primo	
		*Acetobacter diazotrophicus*(MTCC 1226),* Azotobacter chroococcum* (MTCC 3853), *A. vinelandii*(NCIM 2821), *Azospirillum lipoferum*(NCIM 2908), *Rhizobium japonicum*(NCIM 2743)	Agrilife Nitrofix	
		*Azospirillum brasilense*, *Azotobacter vinelandii*, *Bacillus megaterium*, *B. polymyxa*, *Pseudomonas fluorescens*, *Streptomyces albus*	Bactofil A 10	Known for *Pseudomonas*; for other needs investigation
	Phosphate solubilization	*Bacillus subtilis*	Bio Phospho	Needs investigation
		*Bacillus megaterium*	Bio Phos	
		*Pseudomonas striata*(NCIM 2847),* Bacillus polymyxa*(NCIM 2188),* B. megaterium*(NCIM 2087)	P Sol B	Known for *Pseudomonas*
	Zinc mobilization	*T. thiooxidans*NCIM‐5065	Zn Sol B	Needs investigation
	Potash solubilization	*Frateuria aurantia*	Bio Potash	Needs investigation
Soil amendment	Reclaim alkaline soil (oxidizes sulfur and secretes organic acids)	*Thiobacillus thiooxidans*(NCIM 5069)	S Sol B	Needs investigation

**Table 2:** Details of bacterial inoculants used for ecological restoration, as potential targets for studying filamentous phage‐mediated benefits

Bacterial genera	Species	Plant growth‐promoting/bioremedifying traits	Benefits to soil, human, and plants	Filamentous phage (known/needs investigation)	Reference
*Agrobacterium*	*radiobacter*	Provide P and N	Vegetation restoration, Sustainable agriculture	Needs investigation	Belimov, Kojemiakov, and Chuvarliyeva (1995), Belimov Kunakova, et al. (1995), Bashan and Holguin (1997), Singh and Kapoor (1999)
	*tumefaciens*	Degrade pesticides: atrazine, simazine, s‐triazine	Reduce human exposure to xenobiotics and carcinogens; sustainable agriculture		Prashanthi, Sundaram, Jeyaseelan, and Kaliannan (2017)
*Azospirillum*	*amazonense*, *brasilense*, *doebereinerae*	Fix nitrogen, stabilize soil, reduce nutrient load (NH_4_, P) from industrial water	Improve plant growth; reduce toxicant load in wastewater; sustainable agriculture; commercial biofertilizer,	Needs investigation	de‐Bashan, Moreno, Hernandez, and Bashan (2002), Eckert et al. (2001), Volpon, De‐Polli, and Döbereiner (1981), Pindi and Satyanarayana (2012)
	*lipoferum*	Fix nitrogen, provide vitamins, nicotinic acid, IAA, gibberellins	Restoration of arid ecosystems; growth of grasses; sustainable agriculture; commercial biofertilizer		Bashan and Holguin, (1997); Carrillo‐Garcia, Bashan, Diaz Rivera, and Bethlenfalvay (2000); Pindi and Satyanarayana (2012)
*Azotobacter*	*vinelandii*	Fix nitrogen; increase growth of mangrove species	Restoration of mangrove forest; sustainable agriculture	Needs investigation	Bürgmann, Widmer, Sigler, and Zeyer (2003); Kathiresan and Selvam (2006)
	*chroococcum*	Microbe‐assisted phytoremediation of heavy metals (Pb, Zn)	Restoration of mine		Wu, Cheung, Luo, and Wong (2006)
*Acetobacter*	*diazotrophicus*	Fix nitrogen	Vegetation restoration, sustainable agriculture	Needs investigation	Pindi and Satyanarayana (2012)
*Acinetobacter*	*baumannii*PUCM1029; *calcoaceticus*	Fix nitrogen; solubilize phosphate; produce IAA	Environmental restoration; sustainable agriculture	CRAϕ	Rokhbakhsh‐Zamin et al. (2011)
	*radioresistens*	Degrades different pesticides	Environmental restoration; sustainable agriculture	*Acinetobacter baylyi*: CRAϕ	Prashanthi et al. (2017)
	*calcoaceticus*, *lwoffii*, *venetianus*, sp. RTE1.4, sp. HC8‐3S, sp. A3	Degrade organic contaminants (crude oil, halogens, phthalate esters, phenols) in soil and water; reduce nutrient load from wastewater	Environmental restoration; wastewater treatment; sustainable industry	CRAϕ	Fondi et al. (2013); Vamsee‐Krishna, Mohan, and Phale (2006)
*Bradyrhizobium*	*japonicum*	Nitrogen provider	Vegetation restoration, sustainable agriculture	Needs investigation	Rodriguez‐Navarro, Oliver, Contreras, and Ruiz‐sainz (2011)
*Bacillus*	*megaterium*	Fix nitrogen; promote mangrove plant growth; phytoextraction of Pb and Zn	Mangrove and mine restoration; sustainable agriculture	Needs investigation	Kathiresan and Selvam (2006), Pindi and Satyanarayana (2012), Wu et al. (2006)
	*licheniformis*	Fix nitrogen; solubilize phosphate	Mangrove restoration		Rojas, Holguin, Glick, and Bashan (2001)
	*polymyxa*	Solubilize phosphate	Vegetation restoration, sustainable agriculture		Pindi and Satyanarayana (2012)
	*mucilaginosus*	Phytoextraction of heavy metals (Pb and Zn in a mine tailing)	Mine restoration		Wu et al. (2006)
	*Sp*.	Degrade pesticides(trifluralin, endosulfan, Aldrin, lindane)	Environmental restoration, reduction of human exposure to carcinogen and genotoxicants, sustainable agriculture		Prashanthi et al. (2017)
*Clostridium*	*glycolicum*, *collagenovorans*	Bioremediation by volatilization of As (V)	Environmental restoration; reduce human exposure to carcinogens and genotoxicants	CAK1ϕ	Michalke, Wickenheiser, Mehring, Hirner, and Hensel (2000), Meyer, Schmidt, Michalke, and Hensel (2007)
*Erwinia*	*amylovora*HSA6	Mineralize aniline	Environmental restoration; sustainable industry, reduce human exposure to mutagenic and carcinogenic xenobiotics	Known from other members of family (*Escherichia*: M13ϕ, X‐2ϕ, Xϕ, f1ϕ, Ikeϕ, PR6FSϕ, C‐2ϕ, SFϕ, tf‐1ϕ, fdϕ, I2‐2ϕ, If1ϕ, AE2ϕ, Ec9ϕ, HRϕ, ZJ/2ϕ, CUS1ϕ; *Yersinia*:Ypfϕ; CUS2ϕ; *Shigella*:SfXϕ)	Li, Jin, and Yu (2010)
	*carotovora*	Degrades different pesticides (pyrethroidallethrin, β‐cyfluthrin, bifenthrin, cypermethrin, flumethrin and permethrin)			Prashanthi et al. (2017)
*Enterobacter*	*cloacae*	Remediate heavy metals (Cr VI, Pb, Cd and Ni II) and radioactive elements	Environmental restoration, sustainable industry, reduce human exposure to toxicants		Prashanthi et al. (2017), Singh, Walker, Morgan, and Wright (2004), George, Gupta, Gopal, Thomas, and Thomas (2013), Gupta, Saxena, Gopal, and Tilak (2003), Gontia‐Mishra, Sapre, Sharma, and Tiwari (2016)
	*ludwigii*	Assist wheat to tolerate drought	Sustainable agriculture		
	Sp.B‐14	Degrades organopesticides (chlorpyrifos, fonofos, terbufos)	Environmental restoration, sustainable agriculture, reduce human exposure to carcinogens		
	Sp. RNF 267, EG‐ER‐1, KG‐ER‐1	Promote plant growth (coconut palms and maize), fix nitrogen	Sustainable agriculture, increase crop yield		
*Escherichia*	*coli*	Degrades pesticides (atrazine, simazine, s‐triazine), promote plant growth, biocontrol of pathogen by producing bacteriocin (colicins); remediation of heavy metal (Methylmercury); Siderophore production	Reduce exposure of xenotoxic toxicant, carcinogens, Bioremediation, Sustainable agriculture		Prashanthi et al. (2017), Beneduzi, Ambrosini, and Passaglia (2012), Kane et al. (2016), Searle, Méric, Porcelli, Sheppard, and Lucchini (2015)
*Flavobacterium*	sp.	Degrade pesticides (cadusafos, diazinon, dichlorovos, ethoprophos, fenamiphos, fenitrothion, isazofos, isofenphos, isoxathion, malathion, methylparathion, monocrotophos, paraoxon, parathion, phosphomidon and quinalphos); Solubilize phosphate	Environmental restoration, sustainable agriculture, reduce human exposure to carcinogens	Needs investigation	Prashanthi et al. (2017), Pindi and Satyanarayana (2012)
*Frateuria*	*aurentia*	Solubilize potash	Vegetation restoration, sustainable agriculture	Needs investigation	Pindi and Satyanarayana (2012)
*Herbaspirillum*	*seropedicae,*	Fix nitrogen	Vegetation restoration, sustainable agriculture	Needs investigation	Pindi and Satyanarayana (2012)
	*rubisubalbicans*				
	Sp.	Degrade pesticides (trifluralin)	Environmental restoration, sustainable agriculture, reduce human exposure to carcinogens		Prashanthi et al. (2017)
*Micrococcus*	*luteus*	Degrade pesticides (aldrin and lindane)	Environmental restoration, sustainable agriculture, reduce human exposure to carcinogens	Needs investigation	Prashanthi et al. (2017)
	*yunnanensis*SMJ12	Fix nitrogen, solubilize phosphate, produce IAA, siderophores, and 1‐aminocyclopropane‐1‐carboxylate (ACC) deaminase,	Environmental restoration, sustainable agriculture		Mesa et al. (2015)
*Methylobacterium*	sp. CBMB20 and sp. CBMB110	Produce cytokinin and promote sugarcane, tomato, and red pepper crop yield	Sustainable agriculture	Needs investigation	Madhaiyan et al. (2005), Ryu et al. (2006)
*Neisseria*	*flavescens*	Bioremediation of atmospheric hydrocarbons; accelerate phytoremediation of oil contaminants	Environmental restoration, sustainable industry, reduce human exposure to xenotoxicants	Known from genus (*N. gonorrhoeae*: NgoΦ6, NgoΦ7, NgoΦ8, NgoΦ9; *N. meningitides*: MDAΦ, NFϕ; MDAϕ; Nf1‐Aϕ; Nf3‐Aϕ; Nf1‐B1ϕ; B2ϕ; Nf1‐C1ϕ; C2ϕ; C3ϕ; C4ϕ; Nf4‐G2ϕ; G3ϕ; G5ϕ)	Ali et al. (2015)
*Phyllobacterium*	Sp.	Solubilize phosphate; fix nitrogen	Enhance mangrove growth	Needs investigation	Rojas et al. (2001)
*Propionibacteria*	*acnes*	Bioremediation of atmospheric hydrocarbons and accelerate phytoremediation of oil contaminants	Environmental restoration, sustainable industry	B5ϕ	Ali et al. (2015)
	Sp.	Provide nitrogen	Vegetation restoration, sustainable agriculture		Sellstedt and Richau (2013)
*Pseudomonas*	*aeruginosa*UCP1567	Degradation and decolorization of Black B azo dye	Environmental restoration; reduce exposure of xenotoxic toxicants and carcinogens; bioremediation; sustainable agriculture	Known from genus *(P. aeruginosa* *Pf1*ϕ; *Pf2*ϕ; *Pf3*ϕ; Pf4ϕ; Pf5ϕ; Pf7ϕ; Pf‐LESB58ϕ)	Vilar Jr, Cavalcanti, da Silva, Andrade, and Campos‐Takaki (2015)
	*aeruginosa*	Degrade pesticides (HCH, endosulfan)			Prashanthi et al. (2017)
	*cepacia*	Degrade pesticides (endosulfan)			
	*diminuta*	Degrade pesticides (chlorpyrifos, fonofos, terbufos)			
	*fluorescens*	Degrade pesticides (oxadiazon, chlorsulfuron, metsulfuronmethyl); enhance metal uptake in plants			Prashanthi et al (2017), Berg (2009), Vessey, (2003), Chanway, (1997)
	*fluorescens*	Biopesticide for fungus	Sustainable agriculture of cotton		Wang, Wang, and Zhou (2004)
	*maltophilia*	Capable of degrading pesticides: Dicamba	Environmental restoration, sustainable agriculture		Prashanthi et al. (2017)
	*pseudoalcaligenes*	Degrade pesticides (aldrin and lindane)			
	*putida*	Degrade pesticides (Cadusafos, diazinon, dichlorovos, ethoprophos, fenamiphos, fenitrothion, gramoxone, HCH, isazofos, isofenphos, isoxathion, malathion, matancha, methylparathion, monocrotophos, paraoxon, phosphomidon, parathion, quinalphos, vinclozolin)			
	*spinosa*	Degrade pesticides (endosulfan)			Prashanthi et al. (2017)
	*stutzeri*	Degrade pesticides (pyrethroidallethrin, β‐cyfluthrin, bifenthrin, cypermethrin, flumethrin and permethrin)			
	*striata*, *rathonis*	Solubilize phosphate	Revegetation,sustainable agriculture		Pindi and Satyanarayana (2012)
	*vesicularis*, *diminuta*	Enhance growth of algae (*Chlorella*)			Mouget, Dakhama, Lavoie, and Noüe (1995)
	Sp.	Degrade pesticides (2,4‐D, atrazine, chlorotoluron, diuron, isoproturon, linuron, monolinuron, propoxur, simazine, s‐triazine, trifluralin); Pentachlorophenol; produce cytokinin	Environmental restoration; sustainable agriculture and industry; disease resistance in plants		Prashanthi et al. (2017)
*Pseudoalteromonas*	*haloplanktis*	Bioremediation of mercury in aquatic environment	Environmental restoration, ecotoxicity prevention, sustainable industry	Known from other members of family (*Pseudomonas: Pf1*ϕ; *Pf2*ϕ; *Pf3*ϕ; Pf4ϕ; Pf5ϕ; Pf7ϕ; Pf‐LESB58ϕ)	Lohara et al. (2001)
	sp. SCSE709–6	Bioremediation of Cd(II) under high salinity, pH, and temperature			Zhou, Zhang, Ma, Zhou, and Zhang (2013)
*Rhizobium*	*leguminosarum*	Provide nitrogen	Vegetation restoration, sustainable agriculture	Needs investigation	Young et al. (2006); NFTA (1986)
	sp.	Siderophore (carboxylate) production			Mosa, Saadoun, Kumar, Helmy, and Dhankher (2016)
	sp.	IAA producer			Datta and Basu (2000), Bhattacharyya and Pati (2000)
	sp.	Remediation of heavy metal contaminated soils	Reduce exposure of xenotoxicants, carcinogens, bioremediation		Wei and Ma (2010)
		Remediation of As contaminated sites			Reichman (2007), Carrasco et al. (2005)
	*galegae*	Remediation of benzene, toluene, and/or xylene (BTX) from soil	Good growth, nodulation, nitrogen fixation, and a strong rhizosphere occurred in soils contaminated with oil or spiked with *m*‐toluate, a model compound representing BTX		Suominen, Jussila, Mäkeläinen, Romantschuk, and Lindström (2000)
*Ralstonia*	*basilensis*	Capable of degrading pesticides: Atrazine, simazine, s‐triazine	Environmental restoration, sustainable agriculture	Known from genus *(R. solanacearum*: PE226ϕ; RSM1ϕ; RSS1ϕ; RSM3ϕ; RSS0ϕ; p12Jϕ)	Prashanthi et al. (2017)
	*solanacearum*	Bioremediation of atmospheric hydrocarbons; accelerate phytoremediation of oil contaminants			Ali et al. (2015)
	*pickettii*	Solubilize phosphate			Kailasan and Vamanrao (2015)
*Sinorhizobium*	*fredii*	Fix nitrogen	Sustainable agriculture	Needs investigation	Rodriguez‐Navarro et al. (2011)
	*meliloti*	Remediation of polycyclic aromatic hydrocarbons (PAH)	a promising bioremediation strategy for aged PAH‐contaminated soils		Teng et al. (2011)
*Shewanella*	*oneidensis*	Bioremediation of heavy metals and radionuclide Cr(VI), Fe(III), Mn(IV), U(VI), and V(V).	Environmental restoration; sustainable agriculture; reduce human exposure to genotoxicants	SW1ϕ	Fredrickson et al. (2008)
	Sp.	Provide indole; solubilize phosphate	Environmental restoration; sustainable agriculture		Fredrickson et al. (2008)
*Shigella*	*flexneri*	Bioremediation of hydrocarbons and oil from soil	Environmental restoration, sustainable industry	SfXϕ	Duniya, Maikaje, Umar, Ponchang, and Daniel (2016)
*Stenotrophomonas*	*acidaminiphila*	Degrade pesticides (Pyrethroidallethrin, β‐cyfluthrin, bifenthrin, cypermethrin, flumethrin and permethrin)	Environmental restoration; sustainable agriculture	Known from genus *(S. maltophilia*:SMA9ϕ; SHP2ϕ)	Prashanthi et al. (2017)
	*maltophilia*	Tolerate salt; provide resistance in wheat against biotic and abiotic stress			Singh and Jha (2017)
	*maltophilia*	Bioremediation of atmospheric hydrocarbons; accelerate phytoremediation of oil contaminants			Ali et al. (2015)
*Salinicola*	*peritrichatus SMJ30*	Solubilize phosphate; produce IAA, siderophores and 1‐aminocyclopropane‐1‐carboxylate (ACC) deaminase; fix nitrogen	Environmental restoration; sustainable agriculture	Needs investigation	Mesa et al. (2015)
*Thermus*	*scotoductus*	Bioremediation of toxic metals and radionuclides (Cr, Fe, Co, Tc, U, etc.) in the hot spring wastewater or heated nuclear waste streams.	Environmental restoration, sustainable industry, hazardous waste management, wastewater treatment	Known from genus *(T. Thermophiles*: PH75ϕ)	Opperman and van Heerden (2008), Slobodkin (2005)
	*thermophilus*	Root stabilizer	Sustainable agriculture		Singh, Sarma, and Keswani (2017), Stetter (1999)
	HR13	Bioremediation of from hot waste water and anaerobic condition	Environmental restoration, sustainable industry		Gihring and Banfield (2001)
	Sp.	Bioremediation of minerals selenite and tellurite			Slobodkin, Sokolova, Lysenko, and Wiegel (2006), Sokolova et al. (2004), Chiong, Barra, González, and Vásquez (1988), Chiong, González, Barra, and Vásquez (1988)
*Vibrio*	*fischeri*	Degrade pesticides (diuron, chlorotoluron, isoproturon, monolinuron and linuron)	Environmental restoration, sustainable industry	Known from genus *(V. cholera*:493ϕ; CTXϕ; fs1ϕ; fs2ϕ; v6ϕ; Vf12ϕ; Vf33ϕ; VSKϕ; CTXϕ; pre‐CTXϕ; KSF‐1ϕ; VEJϕ; ND1‐fs1ϕ; VCYϕ; VGJϕ; VSKKϕ; VfO4K68ϕ; VfO3K6 (f237)ϕ; VfO3K6ϕ; CTX‐nctϕ)	Prashanthi et al. (2017)
	*sagamiensis*SMJ18	Fix nitrogen, solubilize phosphates, produce IAA, siderophores, and 1‐aminocyclopropane‐1‐carboxylate (ACC) deaminase.	Vegetation restoration, sustainable agriculture		Mesa et al. (2015)
*Xanthomonas*	*axonopodis*	IAA producer	Environmental restoration, sustainable industry	XacF1ϕ	Costacurta, Mazzafera, and Rosato (1998); Ahmad et al. (2014)
*Yersinia*	*frederiksenii*	Degrade pesticides (pyrethroidallethrin, β‐cyfluthrin, bifenthrin, cypermethrin, flumethrin and permethrin)	Environmental restoration, sustainable industry	Known from genus (*Y*.pestis: Ypfϕ; CUS2ϕ)	Prashanthi et al. (2017)

**Table 3:** Details of top ten phytopathogenic bacteria and their associated filamentous phages

Bacterial genus	Species	Pathogenic traits	Filamentous phage	Reference
*Agrobacterium*	*tumefaciens*	Crown gall disease in over 140 species of eudicots	Needs investigation	
*Dickeya*	*dadantii solani*	Soft rot disease	Known from other members of family Enterobacteriaceae (*Escherichia*: M13ϕ, X‐2ϕ, Xϕ, f1ϕ, Ikeϕ, PR6FSϕ, C‐2ϕ, SFϕ, tf‐1ϕ, fdϕ, I2–2ϕ, If1ϕ, AE2ϕ, Ec9ϕ, HRϕ, ZJ/2ϕ, CUS1ϕ ; *Yersinia*:Ypfϕ; CUS2ϕ;*Shigella*:SfXϕ)	Vrancken, Holtappels, Schoofs, Deckers, and Valcke (2013), Starr and Chatterjee (1972)
*Erwinia*	*amylovora*	Fire blight, bacterial wilt; necrosis of tissue		
	*tracheiphila*			
*Pectobacterium*	*carotovorum atrosepticum*	Soft rot and blackleg disease		
*Pseudomonas*	*aeruginosa*PAO1 and PA14	Root infection and plant mortality;	Pf1ϕ; Pf2ϕ; Pf3ϕ; Pf4ϕ; Pf5ϕ; Pf7ϕ; Pf‐LESB58ϕ	Walker et al. (2004)
	*putida*KT2440	Phage reduces bacterial fitness in rhizosphere	Pspu28	Quesada, Soriano, and Espinosa‐Urgel (2012)
	*syringae*	Phytopathogen	Known from *P. aeruginoasa*	Marcelletti and Scortichini (2014)
*Ralstonia*	*solanacearum*	Pathogen: bacterial wilt	PE226ϕ; RSM1ϕ; RSS1ϕ; RSM3ϕ; RSS0ϕ; p12Jϕ	Peeters, Guidot, Vailleau, and Valls (2013)
*Xanthomonas*	*campestris pv. citri*	Citrus canker, bacterial leaf spot	Cf16ϕ; Cf1cϕ; Cf1tϕ; Cf1tvϕ; Lfϕ; Xfϕ; Xfoϕ; Xfvϕ; Cfϕ; Lfϕ; Xoϕ; Xvϕ; Xf2ϕ	Rodriguez et al. (2012), Potnis et al. (2015), Thieme et al. (2005)
	*campestris* pv. *vesicatoria*	Foliage and fruit spot disease	ϕXv	Lin et al., (1994)
	*oryzae*pv. *oryzae*	Bacterial leaf blight in rice	ϕXo	
*Xylella*	*Fastidiosa*	Phytopathogen: Olive quick decline syndrome, bacterial leaf scorch, oleander leaf scorch, coffee leaf scorch, alfalfa dwarf, phony peach disease, Pierce's disease of grapes and citrus variegated chlorosis	Xff1ϕ	Hopkins and Purcell (2002)
*Yersinia*	*pseudotuberculosis*and *enterocolitica*	Infect plants: Affect quality of pasture land and crop plants; cause human disease	Known only from *Y. pestis*: Ypfϕ; CUS2ϕ	Fukushima, Shimizu, and Inatsu (2011)
				Fetherston, Lillard, and Perry (1995)

**Figure 2:**
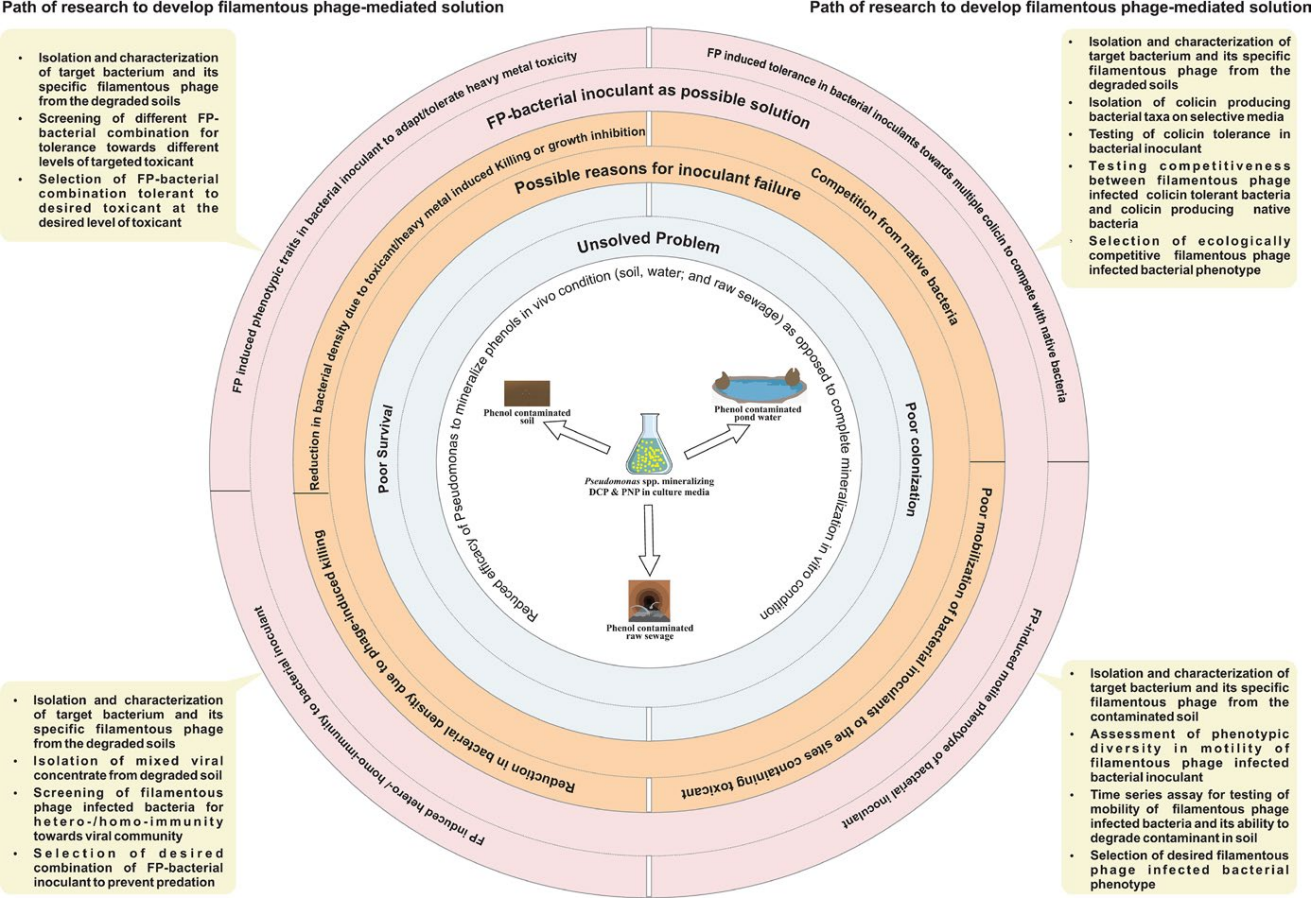
An unsolved problem related to exploiting bioremediation potential of *Pseudomonas* in phenol contaminated field environment highlighting the potential of filamentous phage to provide solutions and path of research to arrive at them (based on Goldstein, Mallory, & Alexander, 1985; Mrozik, Miga, & Piotrowska‐Seget, 2011). FP, filamentous phage.

As a result of advances in molecular and genomic studies, bacterial viruses (bacteriophages or simply phages) have emerged as one of the key elements governing the structure and functioning of microbial communities (Kauffman et al., 2018). Phages play an important role in developing a resilient microbial community (Koskella & Brockhurst, 2014; Silveira & Rohwer, 2016) and in driving adaptation, competition, and antagonism in bacteria and thereby influence the evolution of bacteria and the assembly of microbial community (Rodriguez‐Valera et al., 2009; Mai‐Prochnow et al., 2015; Shapiro, Williams, & Turner, 2016) (Figure 2). However, applying these theories of microbial ecology to ecorestoration has not received due attention.

Among phages, the use of filamentous phages—rod‐shaped single‐stranded circular DNA viruses characterized by a long helical nonenveloped protein coat (King, Lefkowitz., Adams, & Carstens, 2011)—has evolved from answering fundamental questions in biology to developing biotechnological tools (Rakonjac, 2012; Rakonjac, Bennett, Spagnuolo, Gagic, & Russel, 2011). Filamentous phages are of interest to microbial ecologists and biotechnologists because the phages have unique morphological, biological, and genomic features. The life cycle of these phages is marked by chronic infections: The phages multiply continuously within their bacterial host, which releases them into the immediate environment without undergoing cell lysis (Calendar & Inman, 2005; Maniloff, Cadden, & Putzrath, 1981; Rakonjac et al., 2011).Owing to their small and simple genome, filamentous phage can be easily manipulated to display on their surface a variety of peptides or polypeptides, which makes them useful in developing versatile biosensors (Harper & Kutter, 2008; Rakonjac et al., 2011; Table 4). These phages, therefore, have been studied extensively for their morphology and biology (Das, 2014; Krupovic & Forterre, 2015; Marvin, 1998; Opella, Zeri, & Park, 2008; Rakonjac, 2012), for their influence on the physiology of their bacterial hosts (Mai‐Prochnow et al., 2015), for their traditional applications in displaying specified peptides or proteins (Benhar, 2001; Kehoe & Kay, 2005; Willats, 2002), and for their nontraditional applications to develop tools for diagnostic purposes and in nanobiotechnology and synthetic biology for exploring the secretomes of microbes (Rakonjac et al., 2011; Henry, Arbabi‐Ghahroudi, & Scott, 2015; Gagic, Ciric, Wen, Ng, & Rakonjac, 2016; Szekely & Breitbart, 2016; Mai‐Prochnow et al., 2015).

**Table 4:** Comparison of structural, biological, genomic, and functional properties of filamentous phages to that of tailed phages

Property	Character	Filamentous phage	Tailed phages
Classification	Order Family	Not assigned *Inoviridae, Plectrovirus*	*Caudovirales* *Myoviridae, Podoviridae, Siphoviridae*
Morphology	Shape	Long cylindrical	Diverse shape (vary in tail length and type)
	Segmentation	Nonsegmented body	Generally segmented body (head, collar, tail, etc.)
Genome	Genome size respect to body length	Smaller genome size as compared to body length (almost double; 3–9.5 kb)	High genome size
Host	Host	Infect F^+^ bacteria only	No preference for F^+^ or *F* ^−^
Receptor	Receptor	Pilus of the male bacteria	Receptor present on the cell surface
	No. of receptor	Limited numbers (2–4 per cell)	Many (up to few hundreds per host cell)
	Adsorption rate	Adsorption rate constant = 3 × 10^−11^ cm^3^/min	Adsorption rate constant = 2.4 × 10^−9^ cm^3^/min
Infection	Nature	Chronic	Lytic, Temperate
Life cycle	Type	3 types (episomal, constitutively replicating lysogen and inducible lysogen)	2 types (lytic and lysogenic)
Progeny release	Lysis	Does not kill its host	Kills the host
	Progeny release	Progeny released throughout the life span of the host	Progeny released once in host's life
Detection	Plaque formation	Absent or turbid (in some cases)	Clear, turbid or centered
	Observation on plate assay	Difficult	Easy
	Electron#x2010;microscopic assay	Generally neglected as debris or pili of bacteria because of its shape and size	Quite easy to distinguish due to characteristics shape and size

Note

Rakonjac et al. (2011), Mai‐Prochnow et al. (2015).

The growing knowledge of the ecology of filamentous phages from diverse bacterial genera and environmental settings makes them an important biological resource for environmental restoration (Fauquet, Mayo, Maniloff, Desselberger, & Ball, 2005; Rakonjac, 2012; Henry et al., 2015; Szekely & Breitbart, 2016; Mai‐Prochnow et al., 2015). More than sixty filamentous phages have been reported from terrestrial and aquatic ecosystems in temperate and tropical regions (Fauquet, Mayo, Maniloff, Desselberger, & Ball, 2005; Rakonjac, Bennett, Spagnuolo, Gagic, & Russel, 2011). Metagenomic analyses show a high frequency of filamentous phages in such contaminated environments as industrial wastewater and sewage disposal sites, which need ecorestoration (Alhamlan, Ederer, Brown, Coats, & Crawford, 2013; Cantalupo et al., 2011). A recent and exhaustive metavirome study also showed the prevalence of filamentous phages in many diverse environments including soils and sediments, saline water and freshwater, and air and food (Szekely & Breitbart, 2016). Bacteria associated with higher animals, insects, corals, and even people (as gut bacteria) also harbor filamentous phages (Weynberg, Voolstra, Neave, Buerger, & van Oppen, 2015), and they have been reported from bacterial genera associated with ecologically important plant families (Brassicaceae, Poaceae, Rutaceae, and Solanaceae) widely employed in environmental restoration (Berg, Marten, & Ballin, 1996; Tseng, Lo, Lin, Pan, & Chang, 1990). Despite emerging evidence on the prevalence of filamentous phages, current knowledge of the ecology of filamentous phages in soil and plants and of their potential application in restoration continues to be limited because of the challenges in purifying and identifying the phages and in assaying their activities.

Infection by filamentous phages affects the fitness of their bacterial hosts, a feature that can be exploited for securing desired ecological benefits. Mostly, a phage infection enhances the host's ability to combat abiotic and biotic stress, to invade a new habitat, and to partake in the development of microbial communities (Askora & Yamada, 2015; Bille et al., 2005; Derbise & Carniel, 2014; Jian, Xiao, & Wang, 2013; Mai‐Prochnow et al., 2015; Rice et al., 2009; Shapiro et al., 2016; Waldor & Mekalanos, 1996; Webb, Lau, & Kjelleberg, 2004; Yu et al., 2015). However, at times a phage infection lowers the fitness of the host bacterium, which is also beneficial if the bacterial host happens to be a plant pathogen (Ahmad, Askora, Kawasaki, Fujie, & Yamada, 2014; Yamada, 2013). Filamentous phages are useful for manipulating bacteria for environmental applications because the phages are stably produced in their bacterial hosts and are easy to manipulate using genetic and chemical methods—however, they remain underexploited in current practice. A chronic infection by a filamentous phage induces long‐term changes in the host physiology, which is desirable for developing microbial inocula.

Besides a relatively persistent relationship with the bacterial host, filamentous phages also show high host specificity up to the level of a strain, which qualifies them as a stable biomarker of their host (Henry et al., 2015; Lin et al., 1999). For example, ϕLf filamentous phage infects only *Xanthomonas campestris* pv *campestris,*whereas ϕXv filamentous phage infects only *X. campestris*pv. *vesicatoria*: Cross‐inoculation of the filamentous phages and their bacterial hosts did not result in successful infection. Gene III (*gIII*) of the phage encodes a virion‐associated protein (pIII), which shows structural features essential for a phage to be adsorbed on the surface of its host. A hybrid phage of ϕXv with pIII derived from ϕLf could infect *X. campestris*pv. *campestris* successfully but not *X. campestris*pv. *vesicatoria*, showing that the host specificity is governed by *gIII*. As filamentous phages possess unique forms of pIII, such high host specificity makes them useful in tracking and infecting bacterial inocula for environmental applications.

Based on the role of filamentous phages in soil and the ease with which they can be put to biotechnological use, we want to highlight their potential for environmental applications. Specifically, we examined the evidence on (a) the influence of filamentous phages on the ecological and evolutionary potential of their bacterial hosts and (b) the use of filamentous phages in developing biosensing tools for environmental monitoring of microbes and contaminants. We neither provide an in‐depth comparison of different bacterial technologies for environmental restoration nor suggest that the use of filamentous phages along with bacterial inocula can solve every environmental problem. Instead, we highlight the opportunities that filamentous phage present to a practitioner of environmental restoration, especially to design appropriate bacterial inocula and to develop efficient biomonitoring tools. We examine the role of filamentous phages in community ecology and assembly, particularly microbial adaptation, synergism, competition, and antagonism. Finally, we identify priority research areas to realize the potential environmental benefits of filamentous phages and to prevent possible risks in their environmental applications.

## 2 POTENTIAL AREAS FOR THE APPLICATION OF FILAMENTOUS PHAGES IN ENVIRONMENTAL RESTORATION FOR SUSTAINABLE DEVELOPMENT

Environmental restoration is acceptable to ecological economists as a tool for ensuring human well‐being and developing a sustainable society, which is characterized by improved soil health, reduced negative impacts of industrial activity, and lower poverty (Lei, Pan, & Lin, 2016; Martin, 2017; Millennium Ecosystem Assessment, 2005; Sachs & Reid, 2006; Tallis, Kareiva, Marvier, & Chang, 2008). In fact, the policy to encourage environmental restoration proved promising in helping people to escape the poverty trap in China (Cao, Zhong, Yue, Zeng, & Zeng, 2009). Poverty traps represent a vicious circle formed due to a complex interaction between the poverty and environmental degradation, in which “poverty leads to environmental degradation, and environmental degradation then deepens poverty” (Tallis et al., 2008). Poverty forces the native people to engage in unsustainable exploitation of natural resources, which degrades the environment and reduces the resource base for the poor people. Environmental degradation makes the land unproductive, therefore, reduces the income of native people. In this context, ecological restoration programs, which take into account the livelihood of the native people, also restore ecosystem goods and services besides economic and social development. In Changting County of China, the ecological restoration resulted in reduced soil erosion (68.3%), increased vegetation cover (75%), and species number (6 times) accompanied with increased employment (12.4%) and net income (11.2%) of native people (Cao et al., 2009). Policymakers, ecologists, economists, and social scientists were unanimous in emphasizing that ecosystem restoration was vital to achieving at least 7 of the 17 Sustainable Development Goals (SDGs) outlined by the United Nations as part of *Transforming our World: The 2030 Agenda for Sustainable Development* (United Nations, 2015). Those seven goals are as follows. Goal 1: End poverty in all its forms everywhere; Goal 2: End hunger, achieve food security and improved nutrition and promote sustainable agriculture; Goal 3: Ensure healthy lives and promote well‐being for all at all ages; Goal 6: Ensure availability and sustainable management of water and sanitation for all; Goal 8: Promote sustained, inclusive and sustainable economic growth, full and productive employment and decent work for all; Goal 13: Take urgent action to combat climate change and its impacts; and Goal 15, which specifically mentions ecorestoration: “*Protect, restore and promote sustainable use of terrestrial ecosystems, sustainably manage forests, combat desertification, and halt and reverse land degradation and halt biodiversity loss*.”

Soil restoration may involve ex situ or in situ methods to treat and restore degraded lands (Azubuike, Chikere, & Okpokwasili, 2016). In ex situ treatment methods, soil is removed from the affected site and treated either in a bioreactor or on the ground to trigger microbial degradation by manipulating environmental factors such as oxygen, moisture, and nutrients. We may manipulate environmental factors in a bioreactor using an automated controlling system, whereas in the treatment on the ground, we may add organic matter (compost) or fertilizers to the soil, with tillage (land farming) or without tillage (soil biopiles). In situ methods, on the other hand, involve little or no disturbance to soil structure and rely on either natural attenuation by natural physico‐chemical and biological processes or on assisted restoration through enhanced microbial activity. Microbial activity can be enhanced by injecting nutrients, water, chemicals, and even air, using underground pipes, to the contaminated site, that is to the saturated soil zone (biosparging) or to the unsaturated zone (bioventing; Azubuike et al., 2016). Bioventing may be combined with vacuum‐enhanced pumping for treating the contaminants in saturated and unsaturated zones (bioslurping). We may also either introduce specific bacteria to enrich the target bacteria (bioaugmentation) or add specific nutrients to stimulate the activity of targeted bacteria (biostimulation; Malhotra, Mishra, Karmakar, & Sharma, 2017).

In situ and ex situ soil treatments involve costly and labor‐intensive physicochemical methods, and copious use of water puts additional pressure on existing water resources. Also, the use of chemicals to control pathogens adds to the cost, pollutes soil and water, and harms even useful microbes. To avoid these adverse effects, restoration ecologists prefer assisted phytoremediation, which uses plants and their associated microbes to remove or manage the toxicants through such biological processes as rhizofiltration, phytostabilization or biotransformation, biosorption, bioaccumulation or phytoextraction, biodegradation, and biovolatilization (Pilon‐Smits, 2005). Restoration ecologists also select microbial inocula based on their potential to promote plant growth. Microbes promote plant growth by improving soil properties, solubilizing minerals, mobilizing nutrient supply to plants, producing plant growth regulators, and controlling phytopathogens (Rau et al., 2009; Sharma, Mishra, Mohmmed, & Babu, 2011; Sharma, Mohmmed, Mishra, & Babu, 2005; Wubs, Putten, Bosch, & Bezemer, 2016). Thus, the link between below‐ground biodiversity (microbes) and above‐ground biodiversity (plants) is fundamental to ecorestoration. Because contaminated lands harbor fewer forms of life and in smaller numbers, for assisted restoration of vegetation we need to rely on bacteria that not only promote plant growth but are also ecologically competitive (Wubs et al., 2016).

To improve the efficacy of assisted restoration of vegetation, we need to make the outcomes of restoration more predictable and to develop ultrasensitive tools to assess the functionality of sites under restoration (Halme et al., 2013). The tremendous in vitro potential of selected microbes, plants, and a combination of plants and microbes has not been fully realized in the field because these organisms are sensitive to biological toxins, pathogens, contaminants, and such ecosystem challenges as acid or saline soil, inadequate moisture, extreme temperatures, and open soil (Figure 1). Microbial inocula chosen on the basis of their activity in vitro may fail to show similar activity in the field (Figure 2). These challenges may be due to the failure of microbial inocula (poor survival and colonization) or the lack of real‐time biomonitoring tools for tracking the inocula, pathogens, and contaminants—and both can be countered by using filamentous phages to modify the ecophysiology of their bacterial hosts suitably and as ultrasensitive biosensors for real‐time biomonitoring. Filamentous phages carry genes or influence the expression of bacterial genes that help the bacterial hosts to adapt to abiotic and biotic stresses (Shapiro et al., 2016) by developing tolerance to microbial toxins and such abiotic sources of stress in the environment as salinity, desiccation, high temperatures, and high levels of contaminants (Secor et al., 2015; Shapiro et al., 2016; Yu et al., 2015). Filamentous phages may also make their bacterial hosts less virulent or lower our dependency on pesticides to control pathogens, thereby contributing to sustainable restoration (Ahmad et al., 2014; Askora, Kawasaki, Fujie, & Yamada, 2012). Modified filamentous phages may also produce specific peptides or proteins that are useful in monitoring targeted toxins, toxicants, and pesticides in the environment and in holding soil particles together to reduce soil erosion (Curtis, Dunbar, & Macgillivray, 2013; Curtis, Hewitt, & Macgillivray, 2009; Curtis, Macgillivray, & Dunbar, 2011; Goldman et al., 2003; Goldman, Pazirandeh, Charles, Balighian, & Anderson, 2002). In fact, filamentous phages can bridge the gap between the efficacy of microbial activity in vitro and in the field. For example, *Pseudomonas* can mineralize phenols in vitro but not in vivo in the presence of contaminated soil, water, and raw sewage (Goldstein et al., 1985; Mrozik et al., 2011; Figure 2). This failure of *Pseudomonas*to mineralize phenols completely has been attributed to several factors, such as high levels of predation of *Pseudomonas*by phages, stiff competition from native bacteria, greater sensitivity to bacterial toxins, and inadequate mobility of *Pseudomonas*. These factors reduce the viability and survivability of inocula. Filamentous phages have the potential to meet such challenges to the expression of microbial activity in vivo. For example, bacteria infected with filamentous phages proved immune to predation by other phages and to bacterial toxins, were mobile enough to reach the toxicants, and capable enough to degrade them (Addy, Askora, Kawasaki, Fujie, & Yamada, 2012b; Chouikha, Charrier, Filali, Derbise, & Carniel, 2010; Kimsey & Waldor, 1998; Sun & Webster, 1986; Yang et al., 2010). The path of research to develop remediation methods based on filamentous phages that improve microbial action is shown in Figure 2.

**Figure 3:**
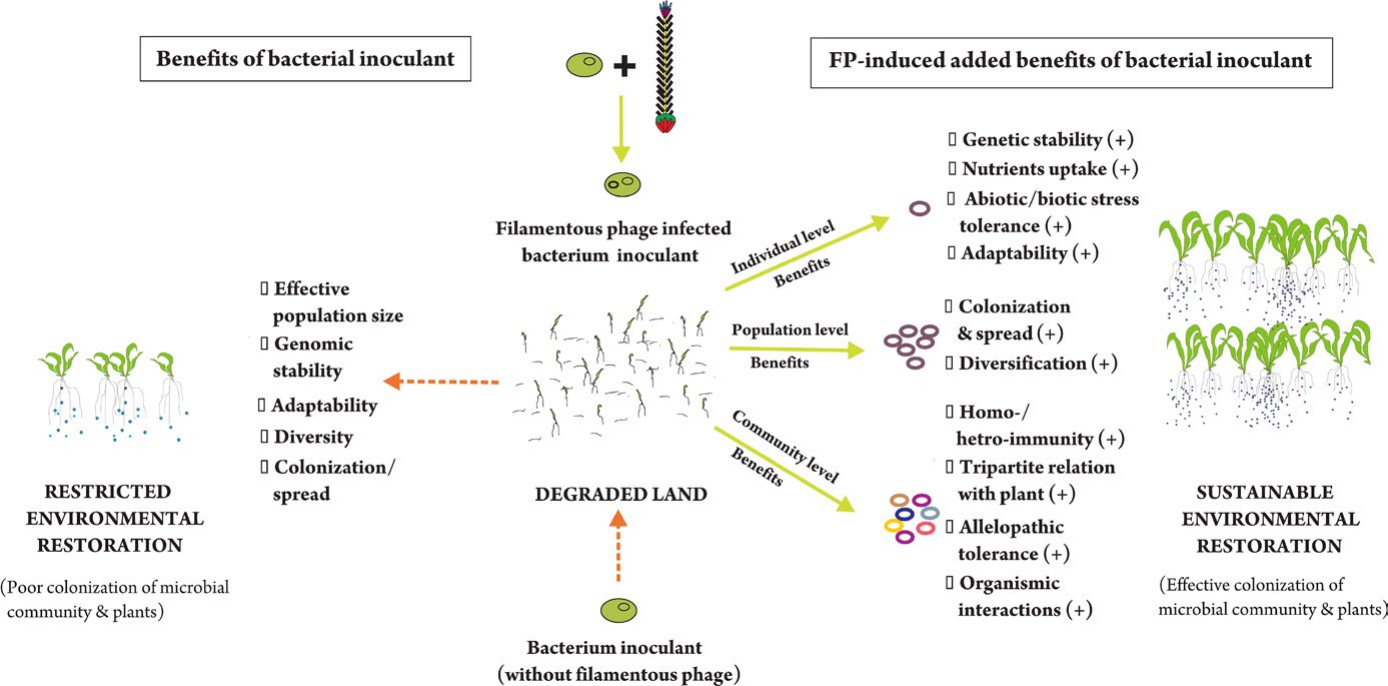
Potential significance of filamentous phages to enhance the ecological and evolutionary potential of the bacterial community to ensure vegetation development at degraded lands

**Figure 4:**
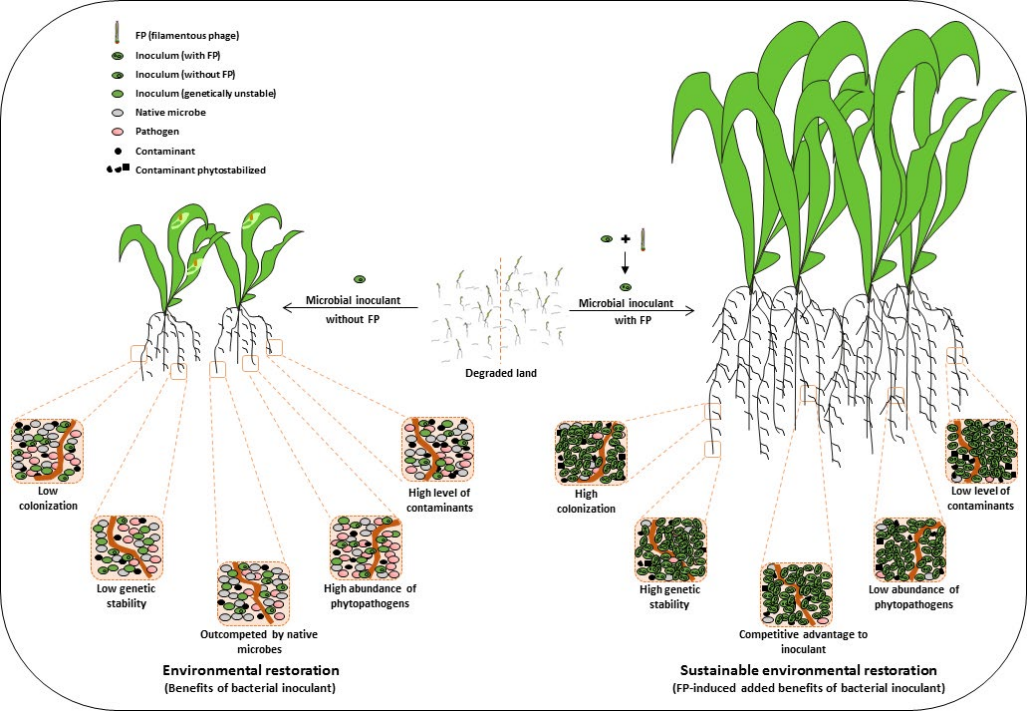
Potential of filamentous phages to assist in the tripartite relation of phage–bacteria–plant to positively influence the upstream effects on plant health, growth, and colonization for ecosystem restoration

Filamentous phages can drive the ecology and evolution of microbial communities (Figure 3). Greater understanding of the role of filamentous phages in promoting host fitness and host diversity and, in turn, their impact on the dynamics of host populations, community composition, and nutrient cycling will help in developing and applying sustainable microbial technologies (Figure 3). Filamentous phages mediate lateral transfer of genes between bacterial strains and drive the evolution of bacterial hosts (Faruque et al., 2005) and also induce phenotypic changes in their bacterial hosts (Table 5), changes that affect the growth of the associated plants directly or indirectly. Based on a meta‐analysis of such studies, we identified potential areas of research to exploit the tripartite relationship between phages, bacteria, and plants (Figure 4). Greater environmental fitness of phage‐infected bacterial hosts strengthens our hypothesis of using filamentous phages that infect plant growth‐promoting rhizobacteria (PGPR) to develop the next generation of bacterial inocula. These inocula consisting of PGPR assist plants in colonizing degraded environments either directly, by promoting root growth, enriching the soil with nutrients, and increasing chelator‐mediated uptake of nutrients, or indirectly, by controlling pathogens and reducing the level of contaminants (Kaur, Pandove, & Gangwar, 2018). However, filamentous phages should also enable PGPR to outcompete native bacteria in colonizing the soil and the rhizosphere. To achieve this goal, we recommend co‐inoculation with filamentous phages and bacteria for ecorestoration (Figures 3 and 4). To this end, we first need to identify the filamentous phages that can bring about the desired changes in the biology and ecology of target bacteria to improve their efficacy as inoculants. Secondly, we need to identify filamentous phages—or modify them—to develop biosensors to track the inocula and the contaminants through time and space. Research in these areas will contribute to making microbial technologies both sustainable and effective.

**Table 5:** Experimental evidences showing influence of filamentous phage on competitiveness of host bacteria

Influence on host bacterial competitiveness	Filamentous phage – host bacterium	Changes in host phenotype	Reference
**Phages in theory of bacterial adaptation:** *As an agent to improve adaptation of bacterial host toward abiotic stresses*			
Increase in tolerance to high temperature (35°C)	Xf2 – *X. campestris*pv. *Oryzae* N5850	• Altered growth pattern (a slow growth in first 60 hr followed by fast growth)	Kamiunten and Wakimoto (1981)
Development of adaptive phenotype due to reduction in rate of cell division and growth rate	M13 – *E. coli* S‐26	• Reduction in growth rate due to increase in mean generation time (25%) and duration of lag phase	Brown and Dowell (1968)
	φRSM (φRSM3, φRSM4) – *R. solanacearum* (MAFF730139, MAFF106611, UW551 )	• Reduction in growth rate by ~60%	Askora, Kawasaki, Usami, Fujie, and Yamada (2009)
	φRSS1, φRSM1 – *R. solanacearum* C319; Ps29		Yamada et al. (2007)
	φM13 – *E. coli* W6		Wan and Goddard (2012)
	φM13‐km – *E. coli* TOP10F	• Reduction in growth rate	Lin et al. (2011)
	M13 – *E. coli* HfrC		Roy & Mitra (1970)
	f327 – *Pseudoalteromonas* sp. BSi20327		Yu et al. (2015)
	M13 – *Escherichia coli* 112‐12		Salivar, Tzagoloff, and Pratt (1964)
	Cf1c – *X. campestris* pv. C*itri*		Kuo, Tan, Su, and Yang (1991)
Tolerant to radiation	φRSS1, φRSM1 – *R. solanacearum* C319; Ps29	• Increase dark coloration and pigmentation	Yamada et al. (2007)
	φRSM (φRSM3, φRSM4) – *R. solanacearum* (MAFF730139, MAFF106611 UW551 )		Askora et al. (2009)
Regulation of host bacterial community under seasonal fluctuation in extreme arctic environment	f327 – *Pseudoalteromonas* sp. BSi20327	• Reduction in cell density and tolerance to NaCl and H_2_O_2_ coupled with increase in motility and chemotaxis (escape from nutrient‐deficient, highly saline environments during arctic winter; and avoid over blooming under H_2_O_2_, nutrient and radiation abundance of arctic summer)	Yu et al. (2015)
Development of freeze‐fracture resistance	fd – *E. coli* HB11	• Increased total lipid content (25%) of outer membrane without affecting relative concentration of phospholipids	Bayer and Bayer (1986)
Provide adaptive fitness to ensure survival in limited‐energy deep sea environment	SW1 – *Shewanella piezotolerans* WP3	• Reduction in swarming motility due to decreased production of lateral flagella with concomitant increase in number of filamentous phages	Jian, Xiao, and Wang (2013)
Increase in adaptation to tolerate and sequester high levels of copper and other heavy metals	Unknown – *Ralstonia pickettii* strains (12D and 12J)	• Horizontal transfer of metal resistance and transporter genes, and zot‐like toxin	Yang et al. (2010)
Increase in tolerance to alkaline pH and salt stress; maintenance of redox and energy state	f1 – *E. coli*	• Induction of phage shock protein response (secretion of pIV secretin); maintenance of PMF‐ and ATP‐dependent protein secretion	Joly et al. (2010)
Tolerant to desiccation	Pf – *P. aeruginosa*	• Increased cellular viscosity, aggregation, and adhesion; promotion of liquid crystalline organization of biofilm matrix	Secor et al. (2015)
Generation of high cellular energy during early growth phase; reduction in survival during acid shock	M13 – *E. coli*	• Upregulation of phosphotransferase; downregulation of acid stress and stationary phase transition genes; impairment of oxidative and acid‐resistance systems	Karlsson, Malmborg‐Hager, Albrekt, and Borrebaeck (2005)
**Phages in theory of microbial competition:** *As an agent to protect the bacterial inoculant from allelopathy effect*			
Development of tolerance to multiple colicins (E1, E2, and E3)	f1 – *E. coli* K38	• Increase in deoxycholate sensitivity, leakage of b‐lactamase, and number of defective *F*‐pili	Boeke, Model, and Zinder (1982)
	f1 – *E. coli* GM1, JM1	• Modifications in tolA and tolB colicin transporter	Sun and Webster (1986)
Provide heteroimmunity	CTXφ – *V. cholerae*	• Divergence of phage repressors and their cognate operators (rstR‐ig‐2)	Kimsey and Waldor (1998)
Provide homoimmunity	YpfΦ – *Y. pestis* biovar *Orientalis* (CO92), Antiqua (IP550‐HC1), Medievalis (IP1865–12))	• Stable integration of YpfΦ genome as multiple tandem repeats into host chromosome providing homoimmunity to phages	Chouikha et al. (2010)
**Phage in theories of microbial colonization and antagonism:** *As an agent to ensure better colonization of host bacteria and control bacterial pathogens*			
Increase in colonization potential to new surfaces by increase virulence, transmissibility, infect wide host range, toxin production, biofilm and aggregation	Nf or MDA – *Neisseria meningitidis* Z2491	Prevalence (90%) of hyper invasive pathogenic strains Development of virulence due to transfer of meningococcal disease associated (MDA) pathogenicity island and zonula occludens toxin (zot)‐like protein	Bille et al. (2005), Joseph et al. (2011)
	Pf4 – *P. aeruginosa* PAO1	Increase in virulence and evolution of super infective form; enhanced stability of biofilms Triggering of biofilm formation with small size hollow colony formation	Rice et al. (2009)
	CTXφ – *Vibrio cholerae* O395	Development of virulent cholera causing strain Acquiring of ability to produce cholera toxin *ctx*AB	Waldor and Mekalanos (1996)
	Xf2 – *X. campestris* pv. *Oryzae* N5850	Increase in virulence (larger size lesions) Increase in EPS production	Kamiunten and Wakimoto (1981)
	Ypfφ – *Yersinia pestis* biovar *Orientalis*	Emergence of highly competitive, virulent (low LD50) plague causing new pathogen with epidemic spread Increase in toxin production, stability of Ypfφ integration in bacterial host chromosome, secretion of phages having ability to infect new hosts; horizontal transfer of toxin genes	Derbise et al. (2007)
	φRSS1 – *R. solanacearum* MAFF 106603 and MAFF 106611	Enhanced virulence of bacterial host leading to an early onset of wilting and spread of infection in tomato Increase in extracellular polysaccharide **(**EPS**)** production, cell surface hydrophobicity, cell aggregation and density; pathogenicity traits (increase in twitching motility and pilin production; early expression of phcA global virulence regulator)	Addy, Askora, Kawasaki, Fujie, and Yamada (2012a)
	VPIφ – *V. cholera* strains (N16961 and 395)	Evolution of potentially pathogenic, nonepidemic strains (non‐O1 and non‐O139) Horizontal transfer of toxin‐coregulated pilus gene (*tcpA*) residing in vibrio pathogenicity island	Li, Kotetishvili, Chen, and Sozhamannan (2003)
	fs2 – *V. cholera* O1	Increase in virulence and reduction in colonizing ability; increase in in vivo production of cholera toxin (CT) and phage CTXf Lateral transfer of *rstC* gene in *V. cholerae* O1 and O139; reduction in type IV fimbrial production and hemagglutination activity; increase in in vivo detachment of cells	Nguyen et al. (2008)
	Cf1c – X. campestris pv. Citri	Evolution of virulent pathogenic variants	Kuo et al. (1991)
	Pf4 – P. aeruginosa PAO1	Increase in colonization potential to new surfaces Triggering of attachment and biofilm formation; development of small colony variants (SCVs)	Webb et al. (2004)
	f1, c2 – Enterobacteria sp.	Development of superinfective forms due to high frequency of mutations Development of small colony variant; loss of cell viability and reduction in rates of RNA and protein synthesis	Kuo, Yang, Chen, and Kuo (2000)
	Pf – *P. aeruginosa*	Increase in transmissibility, pathogenic persister phenotype Increased cellular viscosity, aggregation and adhesion; promotion of liquid crystalline organization of biofilm matrix	Secor et al. (2015)
	φRSS1, φRSM1 (Ff‐type) – *R. solanacearum* C319; Ps29	Enhanced virulence to cause early onset of wilting in tobacco plants	Yamada et al. (2007)
	Pf1 – *Pseudomonas aeruginosa* PAO1	Development of antimicrobial tolerant biofilms Transfer of genes within biofilms due to phage induction	Whiteley et al. (2001)
	PE226 – *R. solanacearum* SL341	Emergence of high virulence to infect wide host range (pepper, tomato and tobacco); Acquiring of zot‐like protein (putative bacterial virulence factor)	Murugaiyan et al. (2010)
	YpfΦ – *Y. pestis* biovar *Orientalis* (CO92), Anti*q*ua (IP550‐HC1), *Medievalis* (IP1865‐12)	Emergence of highly virulent and pathogenic strain by transformation of Y. pseudotuberculosis after horizontal acquisition of filamentous phage YpfΦ over the last 20,000 years	Chouikha et al. (2010)
Enhance host cell aggregation for colonization but reduced virulence	XacF1 – *Xanthomonas axonopodis* pv. *citri*	Reduction in colonization ability, biofilm formation and virulence to cause citrus canker Reduction in swimming, swarming, and twitching motility; low levels of PilA type IV pili; reduction in cell adhesion due to EPS production; slow growth rate	Ahmad et al. (2014)
	φRSM1 and φRSM3 – *R. solanacearum*MAFF 106603 and MAFF 106611	Loss of virulence to show wilting symptoms in tomato Reduction of twitching motility, type IV pili, β‐1,4‐endoglucanase activity, EPS production; and expression of pathogenicity genes (*egl, pehC, phcA, phcB, pilT*, and *hrpB*)	Addy et al. (2012b)
	φRSM (φRSM3, φRSM4) – *R. solanacearum* (MAFF730139, MAFF106611 UW551 )	Extreme reduction in pathogenic potential to cause wilting in tomato Increase cell aggregation and colony size	Askora et al. (2009)
**Phages in theory of community assembly and evolution:** *As an agent to influence evolutionary potential of bacterial inoculant and trigger microbial community development*			
Reduction in conjugative ability and plasmid transfer function	f1 – *E. coli* K38	• Number of defective *F*‐pili	Boeke et al. (1982)
	Ike – *E. coli* K12 RM98	• Alteration in cell membrane proteins	Iyer , Darby, and Holland, (1976)
Diversification in Isogenic population	M13 – *E. coli*	• Induction of high variability in individual viral production than other phenotypic traits in isogenic bacterial population	De Paepe, De Monte, Robert, Lindner, and Taddei et al. (2010)
Maintenance of conjugation rate and spread of antibiotic resistance within population	φM13 – *E. coli* W6	• Reduction in conjugation efficiency by ~10%	Wan and Goddard (2012)
	φM13‐km – *E. coli* TOP10F	• Reduction in average number of pili; decrease in conjugation rate with increase in pfu/ml	Lin et al. (2011)
Evolution and Development of superinfective forms and virulent pathogenic variants due to high frequency of mutations	Cf1c – *X. campestris* pv. *Citri*	• Variation in gene structure and sequence	Kuo et al. (1991)
	f1, c2 – Enterobacteria sp.	• Loss of cell viability and reduction in rates of RNA and protein synthesis	Kuo et al. (2000)

## 3 PLANT‐ASSOCIATED BACTERIA IN ECORESTORATION: CURRENT STATUS AND OPPORTUNITIES FOR FILAMENTOUS PHAGES

### 3.1 Bacteria as restoration inocula: Potential targets for modulation by filamentous phages

The bacteria used in commercial formulations as inocula for such environmental applications as improving soil health and promoting plant growth serve as potential targets for research involving filamentous phages. Table 1 lists such bacterial taxa and their specific beneficial traits (solubilizing and mobilizing nutrients, ameliorating soils, and controlling phytopathogens). At least 47 such commercial formulations are available in the global market. The single‐function formulations use a single bacterial strain or a consortium of bacterial species for improving soil health and promoting plant growth (Table 1), whereas multifunctional formulations rely either on a consortium of bacteria with different activities or a single bacterial strain with multiple desired traits. The most common bacterial genera in these formulations include *Acetobacter*, *Acidithiobacillus*, *Acidovorax*, *Azospirillum*, *Azotobacter*, *Bacillus*, *Bradyrhizobium*, *Chromobacterium*, *Delftia*, *Frateuria*, *Lactobacillus*, *Pseudomonas*, *Rhizobium*, *Rhodobacter*, and *Thiobacillus*. To make these formulations more effective, we suggest that these genera be used as potential targets for exploring the benefits of filamentous phages, although filamentous phages for *Pseudomonas* have already been reported.

Some genera also show potential to remediate soil and water contaminated with inorganic and organic toxicants (Table 2) and therefore form another set of target bacteria for phage research. For example, *Azotobacter*, *Bacillus*, *Clostridium*, *Enterobacter*, *E. coli*, *Pseudoalteromonas*, *Rhizobium*, *Shewanella*, and *Thermus* help in dealing with different heavy metals (Table 2). *Agrobacterium*, *Acinetobacter*, *Bacillus*, *Erwinia*, *Enterobacter*, *E. coli*, *Flavobacterium*, *Herbaspirillum*, *Micrococcus*, *Pseudomonas*, *Ralsotonia*, *Stenotrophomonas*, *Vibrio*, and *Yersinia* degrade different classes of pesticides (Umadevi, Ayyasamy, & Rajakumar, 2017). However, *Acinetobacter*, *Erwinia*, *Neisseria*, *Propionibacterium*, *Pseudomonas*, *Ralstonia*, *Rhizobium*, *Shigella*, and *Stenotrophomonas* degrade diverse organic contaminants including aromatic amines, azo dyes, phenols, benzenes, toluenes, xylenes, oils, polyaromatic hydrocarbons, halogens, and phthalate esters (Table 2).

Besides their potential in environmental remediation, some bacterial genera also promote plant growth through such means as mineral solubilization, nitrogen fixation, phytohormone production, and antagonism toward pathogens (Table 2). These bacteria can enhance plant growth because they can solubilize insoluble phosphates. Such phosphate‐solubilizing bacteria (PSBs) include *Acinetobacter*, *Agrobacterium*, *Bacillus*, *Flavobacterium*, *Lysinibacillus*, *Microbacterium*, *Micrococcus*, *Paenibacillus*, *Pseudomonas*, *Ralstonia*, *Salinicola*, *Serratia*, *Shewanella*, and *Vibrio*, and some of them may also fix nitrogen, namely *Agrobacterium*, *Azospirillum*, *Azotobacter*, *Acinetobacter*, *Bacillus*, *Bradyrhizobium*, *Rhizobium*, *Sinorhizbium*, *Ensifer*, *Mesorhizobium*, *Herbaspirilum*, *Micrococcus*, *Phylobacterium*, *Salinicola*, and *Vibrio*.Some bacteria produce indole acetic acid (IAA), a plant growth regulator essential for plants to colonize degraded sites. These IAA‐producing bacteria include *Azospirillum*, *Acinetobacter*, *Microbacterium*, *Micrococcus*, *Rhizobium*, *Salinicola*, *Vibrio*, *Xanthomonas*, and *Vibrio*. Some bacteria are antagonistic to phytopathogens; these include *Bacillus*, *Micrococcus*, *E. coli*, *Paenibacillurs*, *Rhizobium*, *Pseudomonas*, *Salinicola*, and *Vibrio*.Therefore, research on filamentous phages to infect these bacterial genera deserves higher priority.

### 3.2 Bacteria with known filamentous phages potentially useful in restoration

Filamentous phages have been reported from many bacterial genera potentially useful in ecorestoration. These genera include *Acinetobacter*, *Clostridium*, *Enterobacteria*, *Neisseria*,* Propionibacterium*,* Pseudoalteromonas*,* Pseudomonas*,* Ralstonia*,* Shewanella*,* Shigella*,* Stenotrophomonas*, *Thermus*,* Vibrio*,* Xanthomonas*, *Xylella*, and *Yersinia* (Addy et al., 2012b; Ahmad et al., 2014; Derbise et al., 2007; Jian et al., 2013; Kuo et al., 2000; Waldor & Mekalanos, 1996; Whiteley et al., 2001; Yu et al., 2015; Table 2). These genera include species that cause diseases in plants and animals, show bioremediation activity, and promote plant growth. The filamentous phage‐mediated ecological fitness of host bacteria has been investigated in selected species for pathogenicity, survival, colonization, multiplication, and distribution in a given ecological niche (Table 5). However, other species that may be potentially useful in bioremediation and restoration of vegetation are yet to be fully exploited.

Most bacterial genera also include species, which have association with plants. For example, *Acinetobacter*, *Enterobacter*, *Pseudoalteromonas*, *Pseudomonas*, *Ralstonia*, *Shewanella*, *Stenotrophomonas*, *Vibrio*, *Xanthomonas*, and *Xylella* represent predominant endophytes, rhizobacteria, or both, with beneficial effects on plant growth (Bhattacharyya & Jha, 2012; Borriss, 2011; Chandra & Singh, 2016; Kobayashi & Palumbo, 2000; Tilak et al., 2005). *Neisseria*, *Propionibacterium*, *Ralstonia*, and *Stenotrophomonas* form hydrocarbon‐degrading bacterial communities that inhabit the phyllosphere of plant species that have been widely used for phytoremediation of air and soil contaminated with hydrocarbons (Al‐Awadhi et al., 2013; Al‐Mailem et al., 2010). *Pseudoalteromonas shioyasakiensis* and *Vibrio sagamiensis* SMJ18 are important members of endophytic bacterial populations that inhabit *Spartina maritima*, a species of cordgrass that accumulates heavy metals and is found in most of the polluted estuaries worldwide (Mesa et al., 2015). Of these bacteria, *Pseudomonas* spp. have been widely exploited for bioremediation. *Pseudomonas*also produces diverse molecules to promote plant growth: for example, *P. fluorescens* produces siderophores to promote the growth of plants; *P. chlororaphis* produces phenazine, an antibiotic to control fungal pathogens; and *P. aurantiaca* secretes di‐2,4‐diacetylfluoroglucylmethane, an antibiotic to control Gram‐positive bacterial pathogens.

In fact, species of these bacterial genera have shown their potential in bioremediation and they have been reported from contaminated sites that need to be restored. For example, *Acinetobacter* spp. (*A. calcoaceticus* MM5, *A. lwoffii* ISP4, *A. venetianus*, *Acinetobacter* sp. RTE1.4, *Acinetobacter* sp. HC8‐3S, and *Acinetobacter* sp. A3) degrade such aromatic contaminants as crude oil, halogens, phthalate esters, and phenols in soil and water (Fondi et al., 2013; Vamsee‐Krishna et al., 2006). *Acinetobacter* spp. have also been employed for treating industrial wastewater (Liu et al., 2016), and a protocol has also been developed for their mass multiplication. Strains of *Acinetobacter*, *Neisseria*, *Xanthomonas*, and *Pseudomonas* dominate in a petroleum‐degrading consortium purified from contaminated soils in China (Xu et al., 2011). *Thermus* spp. (*T. scotoductus*, *T. thermophilus* DSM 579, and *T. aquaticus* DSM 625) favor terrestrial hot springs but *T. thermophilus* HB8 has also been reported in organic waste, sewage sludge compost, thermogenic compost, cattle manure, and garden waste (Fujio & Kume, 1991; Marteinsson, Birrien, Raguenes, Costa, & Prieur, 1999). These bacterial genera and species have a high potential for processing and bioremediation of wastes even at temperatures as high as 65–84°C.

Some bacterial genera infected with known filamentous phages also show features useful for ecorestoration (bioremediation, promotion of plant growth, and development of vegetation). For example, *Clostridium*is one of the common bacterial genera reported from the rhizosphere (Dinesh et al., 2015): *C. glycolicum* and *C. collagenovorans* volatilize As (V) (Meyer, et al., 2007; Michalke, et al., 2000). Singh et al. (2004) reported the potential of *Enterobacter cloacae* B2‐DHA to bioremediate heavy metals (Cr VI, Pb, Cd, and Ni II) and radioactive elements and that of *Enterobacter* B‐14 to biodegrade organophosphate pesticides in contaminated soils. A bacterial consortium comprising *Pseudomonas*, *Acinetobacter*, and *Neisseria* mineralizes DDT (Carrillo‐Pérez, Ruiz‐Manríquez, & Yeomans‐Reina, 2004) and a consortium comprising *Acinetobacter faecalis*, *Neisseria elongate*, and *Staphylococcus* sp. efficiently degrades crude petroleum oil (Mukred, Hamid, Hamzah, & Yusoff, 2008). *Pseudoalteromonas* has proved useful in bioremediation of substrates contaminated with inorganic and organic pollutants. For example, *Pseudoalteromonas* sp. SCSE709‐6 from the deep sea showed a great capacity (96%) to remove Cd(II) at varying temperatures and varying levels of pH and salinity (Zhou, et al., 2013). *Pseudoalteromonas*TG12 solubilizes Fe, accumulates different metals (Gutiérrez, Shimmield, Haidon, Black, & Green, 2008), and degrades alkanes and cycloalkanes (Dubinsky et al., 2013). *Pseudoalteromonas* and *Vibrio* purified from sediments in San Diego Bay degraded hydrocarbons and a toxic organic pollutant (phenanthrene or chrysene; Coelho, Rivonkar, Bhavesh, Jothi, & Sangodkar, 2003; Melcher, Apitz, & Hemmingsen, 2002), and *P. haloplanktis* from Minamata Bay, Japan, was investigated for its resistance to mercury (Lohara et al., 2001) and as a model organism for genetic manipulation in bioremediation studies (Kivelä, Madonna, Krupovìč, Tutino, & Bamford, 2008). *Pseudomonas* spp. have received attention for their bioremediation potential to deal with diverse organic contaminants. For example, *P. alcaligenes*, *P. mendocina*, and *P. putida* degrade polycyclic aromatic hydrocarbons (e.g., toluene); *P. veronii* degrades different simple aromatic compounds; *P. resinovorans* degrades aromatic heterocyclic compounds (e.g., carbazole and quinoline); *P. stutzeri* KC degrades haloalkanes (e.g., carbon tetrachloride); and *P. pseudoalcaligenes* uses cyanide as a nitrogen source. In populations of *Ralstonia pickettii*12D and 12J, infection by filamentous phages makes their bacterial hosts more adaptable to heavy metals by increasing horizontal gene transfer in the bacterial populations (Yang et al., 2010). The metabolic versatility of* Shewanella* also makes it a member important in cycling metals and organic matter (Fredrickson et al., 2008). For example, *S. oneidensis* has the potential to remediate substrates contaminated with Cr(VI), Fe(III), Mn(IV), U(VI), and V(V). The role of *Shewanella* in nutrient cycling becomes important because Mn is the second most abundant metal in the earth's crust, an essential trace element for all living organisms, and also influences the cycling of other elements. *Thermus scotoductus* is widely used for immobilizing toxic metals and radionuclides (Cr, Co, Fe, Tc, U, etc.) from wastewater from hot springs or heated streams of nuclear waste (Brim, Venkateshwaran, Kostandarithes, Fredrickson, & Daly, 2003; Kashefi & Lovley, 2000; Kieft et al., 1999; Opperman & van Heerden, 2008; Slobodkin, 2005). *Thermus oshimai* can remove heavy metals (Poli et al., 2009), and *Thermus* sp. removes selenite and tellurite (Chiong et al. 1988; Slobodkin et al. 2006; Sokolova et al., 2004).

*Enterobacter*, *Pseudoaltermonas*, and *Vibrio*species are not only significant for bioremediation but also for promoting plant growth. *Enterobacter* sp. RNF 267 promotes the growth of coconut palms (*Cocos nucifera*) and maize (George, 2013), and inoculation of green gram (*Vigna radiata*) with *Enterobacter* EG‐ER‐1 and KG‐ER‐1 together with *Bradyrhizobium* sp. increased nodulation (Gupta et al. 2003). *P. shioyasakiensis* and *V. sagamiensis* SMJ18 tolerate not only salt and heavy metals (As, Cu, and Zn) but also show multiple traits that promote plant growth: They can fix nitrogen; solubilize phosphates; and produce IAA‐, siderophores, and ACC (1‐aminocyclopropane‐1‐carboxylate deaminase). *Spartina maritima* inoculated with *V. sagamiensis* SMJ18 shows more efficient photosynthesis, greater intrinsic water‐use efficiency, and lower metal uptake—which is why the combination of *V. sagamiensis* and *S. maritima* has been recommended for ecorestoration of polluted estuaries.

As most of the above‐mentioned bacterial genera can be commercialized and filamentous phages of these genera are known (Table 2), co‐inoculation with bacteria and filamentous phages needs to be tested for environmental use (Figures 3 and 4).

**Figure 5:**
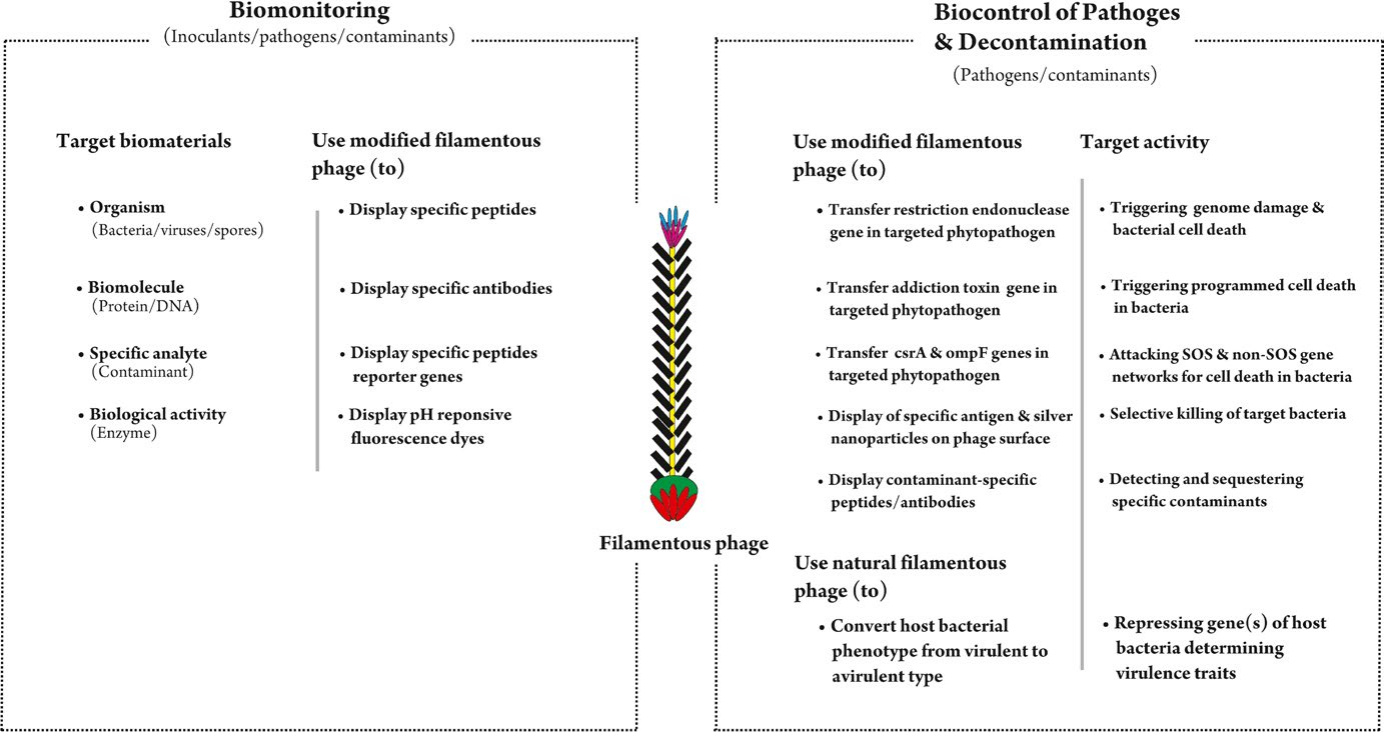
Potential significance of filamentous phages to develop efficient biomonitoring system for tracking and management of targeted inoculated strain, pathogens, and contaminants

### 3.3 Phytopathogenic bacteria particularly useful in ecorestoration as targets of research on filamentous phages

Filamentous phages of bacterial phytopathogens also provide an opportunity to improve assisted phytoremediation as part of ecorestoration (Table 3; Figure 4). Filamentous phages have been characterized for six of the world's ten most serious bacterial phytopathogens. These six pathogens belong to four genera, namely *Pseudomonas*, *Ralstonia*, *Xanthomonas*, and *Xylella* (Mansfield et al., 2012). For three more phytopathogenic bacteria, namely *Dickeya*, *Erwinia*, and *Pectobacterium*, filamentous phages have been reported from related bacterial genera. Filamentous phages of other phytopathogenic genera, namely *Acinetobacter*, *Clostridium*, and *Pseudoalteromonas*, are also relevant because these genera also have species that are pathogenic to plants used for remediation. However, filamentous phages of *Propionibacterium* and *Yersinia*, opportunistic pathogens of the human body, are also important because these pathogens use plants as temporary hosts and therefore need to be controlled. Filamentous phages determine the pathogenicity of some bacteria, which is why the biochemical and molecular mechanisms of phages–bacteria interactions can be the key to developing biocontrol methods for phytopathogens. Filamentous phages of avirulent bacterial strains would also be useful as competitors to the filamentous phages of virulent bacterial strains.

## 4 FILAMENTOUS PHAGES TO BOOST ENVIRONMENTAL COMPETITIVENESS OF BACTERIAL INOCULA

In many bacteria, filamentous phages influence the expression of phenotypic traits and trigger the appropriate ecophysiological mechanisms that help the bacteria to adapt better to sources of stress in the environment. Sometimes, a phage infection triggers a high level of cellular organization to prevent the host cells from being exposed to the sources of stress in the outside environment (Table 5). Experimental evidence shows that filamentous phages drive the ecological success of their host bacteria in a given niche. Such mechanisms have been investigated in *Acinetobacter*, *Clostridium*, *Enterobacteria*, *Neisseria*,* Propionibacterium*,* Pseudoalteromonas*,* Pseudomonas*,* Ralstonia*,* Shewanella*,* Shigella*,* Stenotrophomonas*, *Thermus*,* Vibrio*,* Xanthomonas*, *Xylella*, and *Yersinia* (Addy et al., 2012b; Ahmad et al., 2014; Derbise et al., 2007; Jian et al., 2013; Kuo et al., 2000; Waldor & Mekalanos, 1996; Whiteley et al., 2001; Yu et al., 2015). As discussed earlier, these genera are also important in bioremediation and in promoting plant growth. Filamentous phages therefore have the potential to improve the ecological and evolutionary potential of bacterial inocula so that the bacteria survive environmental stress, evolve in the changing environment, and contribute to the growth of plants (Figure 3).

### 4.1 Phages and microbial adaptation

Filamentous phages influence the growth of their bacterial hosts to increase the adaptive potential of the hosts (Table 5). For example, *E. coli*(112‐12, S‐26, W6), *Pseudoalteromonas* sp. (f327), *Ralstonia solanacearum* (C319, Ps29), and *Xanthomonas campestris*(pv. N5850) infected with filamentous phages show slower growth and greater adaptability to stress (Brown & Dowell, 1968; Kamiunten & Wakimoto, 1981; Salivar, et al., 1964; Wan & Goddard, 2012; Yamada et al., 2007; Yu et al., 2015). The bacterial host survives either because its growth is put on hold until favorable conditions return or because it gets adequate time to activate appropriate mechanisms to combat stress from abiotic sources. Therefore, *E. coli* infected with a filamentous phage shows reduced growth but greater resilience to changes in the environment; the noninfected and fast‐growing bacterial hosts, on the other hand, show a high metabolic rate and use up the energy for growth, thereby becoming more susceptible to stress (Tamman, Ainelo, Ainsaar, & Hõrak, 2014; Tuomanen, Cozens, Tosch, Zak, & Tomasz, 1986; Yu et al., 2015). Such phage‐mediated phenotypic and ecophysiological changes in bacterial hosts are immensely useful in ecorestoration. These changes in bacterial inocula will help the bacterial hosts to adapt to stress from abiotic sources, to maintain effective bacterial populations, and to perform their desired ecological functions (Arora, Tiwari, & Singh, 2014; Gopalakrishnan et al., 2015; Lahav, 1962; Malusá, Sas‐Paszt, & Ciesielska, 2012).

Other evidence shows that filamentous phages enhance the adaptive potential of bacterial hosts by influencing specific phenotypic traits or biological processes (Table 5). In *E. coli* HB11, infection from fd phage increases the total lipid content, which helps the host to resist freeze‐fracture stress (Bayer & Bayer, 1986). Cells of *Shewanella piezotolerans* WP3 infected by the SW1 phage show fewer lateral flagella and poor swarming motility (Jian et al., 2013), which enables the bacteria to survive the limited‐energy environment. In *Ralstonia pickettii*(12D, 12J), filamentous phages mediate horizontal gene transfer and enable the bacterial host to adapt to a high level of Cu and other heavy metals in lake sediment (Yang et al., 2010). M13‐km phage infection of *E. coli* TOP10F decreases conjugation and prevents the spread of antibiotic resistance genes in bacterial populations (Lin et al., 2011); M13 infection of *E. coli*K12 leads to loss of lipopolysaccharide, which makes the strain more susceptible to actinomycin D (Roy & Mitra, 1970b); and f1‐infection of *E. coli* enables the bacterial cell envelope—by means of phage shock proteins in the bacterial host—to tolerate stress in many forms (high pH, high concentration of salts, etc.; Joly et al., 2010).

Besides these mechanisms, filamentous phages also trigger highly structured organization of bacterial populations or communities (biofilm, for example), which protects their members from several sources of environmental stress (Table 5): Suspensions of *Pseudomonas aeruginosa* cells infected with Pf filamentous phage become more viscous, which helps the host cells to aggregate and adhere together to form a biofilm, which enables the assembly to survive desiccation and offers protection from aminoglycoside antibiotics and toxic chemicals (Secor et al., 2015; Webb et al., 2004). A biofilm consisting of multiple species is a cross‐species communication network that enables the constituent species to use nutrients including C more effectively when they are in short supply (Flemming et al., 2016). Based on the evidence discussed here, we suggest that (a) filamentous phages of target bacteria be isolated and analyzed to develop ecologically competitive bacterial inocula and (b) bacteria used in commercial inocula and potential PGPR be used as hosts to isolate suitable filamentous phages and the adaptive potential of such phages–bacteria co‐inoculation be tested.

### 4.2 Phages to make beneficial bacteria more competitive

Bacteria that form the inocula used for remediation may tolerate stress from abiotic sources but they also need to overcome biotic adversaries, especially the well‐adapted native microbial species (Amarger, 1981; Barnet, 1980; Bashan, 1998; Díaz‐Ramírez, Escalante‐Espinosa, Schroeder, Fócil‐Monterrubio, & Ramírez‐Saad, 2013; Gopalakrishnan et al., 2015; Vincent, 1954). Some researchers believe that stressful habitats favor toxin producers, which invade such habitats and outcompete the toxin‐susceptible strains (Hibbing, Fuqua, Parsek, & Peterson, 2010; Majeed, Gillor, Kerr, & Riley, 2011; Riley & Gordon, 1999). In fact, the markedly lower biodegradation efficacy of bacterial inocula in vivo than that in in vitro has been attributed to their sensitivity to toxins produced by native bacteria (Goldstein et al., 1985; Mrozik et al., 2011).

Infection of *E. coli* (K38, GM1, JM1) and *V. cholerae* by filamentous phages not only increases the ability of the hosts to tolerate toxins but also makes the hosts resistant to infection by other (homo‐ or heterologous) phages. In *E. coli*, infection by f1 filamentous phage leads to irreversible changes in the membrane protein that serves as a common receptor for colicin and phages (Table 5; Boeke et al., 1982; Sun & Webster, 1986; Zinder, 1973). Therefore, we propose the use of filamentous phages to develop ecologically competitive bacterial inoculants that can tolerate toxins and resist other phages in the soil. In *E. coli*K38, infection by filamentous phage triggers a phenotypic change in the membrane, which makes it more sensitive to deoxycholate, promotes leakage of β‐lactamase, and increases the number of defective pilli on the host cell. Due to these changes, *E. coli*K38 tolerates colicins but shows a reduced frequency of conjugation (Boeke et al., 1982). Cells of *Vibrio cholerae* infected with CTXф develop heteroimmunity against lambdoid phages and show a divergence in phage repressors and their cognate operators (rstR‐og‐2; Kimsey & Waldor, 1998). These changes confer a competitive advantage on the infected cells in countering attack by other bacterial species (Davies & Davies, 2010; Feldgarden & Riley, 1998). Thus, filamentous phages have the potential to protect their hosts from biotic sources of stress as well, both chemical and viral, which are common features of the ecosystem in which the inoculants find themselves.

### 4.3 Phages to improve colonizing abilities of bacteria

Bacterial inocula used in remediation should not only survive, by competing successfully with other microbes, but also thrive, colonizing the contaminated sites to provide the desired ecological benefit. Bacteria can become virulent after infection by filamentous phages: Such virulent bacteria can then invade and colonize a particular niche (a habitat or an organism; Table 5). The most noteworthy examples of such bacterium–phage association include *Neisseria meningitidis*and Nf/MDA (Bille et al., 2005), *Pseudomonas aeruginosa* and фPf4 (Rice et al., 2009), *Ralstonia solanacearum* and φRSS1 (Addy et al., 2012b), *Vibrio cholerae* and CTXø (Waldor & Mekalanos, 1996), *Xanthomonas campestris* and Xf2 (Kamiunten & Wakimoto, 1981), and *Yersinia pestis* and YpfΦ (Derbise & Carniel, 2014).

In these bacterial hosts, filamentous phages either carry the virulence gene(s) (Addy et al., 2012b; Derbise & Carniel, 2014; Waldor & Mekalanos, 1996) or regulate the expression of virulence factors (Addy, Askora, Kawasaki, Fujie, & Yamada, 2012a, b; Ahmad et al., 2014). For example, *N. meningitidis*and *Y. pestis* are transformed into virulent strains capable of causing epidemics after receiving the toxin gene from Nf or MDA and from YpfΦ phage, respectively (Bille et al., 2005; Derbise et al., 2007). The filamentous phage CTXΦ transfers to *V. cholerae*O395 the gene ctxAB, which encodes the cholera toxin (Waldor & Mekalanos, 1996). Other strains of *V. cholerae*, namely N16961 and 395, develop into *potential* pathogens after they receive the gene vibrio pathogenicity island (VPI) from the filamentous phage VPIΦ (Li et al., 2003): That potential is realized if a helper phage, namely fs2, infects *V. cholerae*O1 thereby converting the host into a virulent strain. The phage‐mediated transfer of *rstC*gene into *V. cholerae*O1 increases the production of the cholera toxin and triggers the multiplication of the resident CTXΦ phage, thus making the host highly virulent (Nguyen et al., 2008).

Filamentous phages can also convert pathogens into their superinfective forms by changing the phenotypic traits associated with virulence. For example, Xf2 infection converts *Xanthomonas campestris* pv *oryzae*N5850 into a highly virulent phytopathogen by increasing the production of extracellular polysaccharide (Kamiunten & Wakimoto, 1981). Virulent pathogenic variants of *X. campestris*pv *citri*evolve after infection by CF1c phage (Kuo et al., 1991), and infection by the phage PE226 transfers into *Ralstonia solanacearum*SL341 the genes responsible for producing toxins and thus widens the host range of the pathogen (Askora, Kawasaki, Usami, Fujie, & Yamada, 2009). Similarly, *Pseudomonas aeruginosa*PAO1 evolves into a superinfective phenotype after infection with the phage Pf4 (Rice et al., 2009; Webb et al., 2004). Other plant‐associated bacteria, such as *E. coli*(Bayer & Bayer, 1986), *Enterobacteria*(Kuo et al., 2000), *P. aeruginosa*(Secor et al., 2015), and *R. solanacearum*(MAFF106603 and MAFF106611; C319 and Ps29), also evolve into infective phenotypes if they are infected by their specific filamentous phages (Addy et al., 2012b; Yamada et al., 2007; Table 5). Although *E. coli* and *Enterobacter* are part of the microbiome of the human gut, recent research has shown that these bacteria are also native soil bacteria, which promote plant growth and remediate the environment. For example, *E. coli* has been reported from soils from seven geo‐climatic zones of India, and inoculating *Zea mays* with *E. coli* enhances nutrient uptake and plant growth (Nautiyal & Shono, 2010). In fact, plants exert niche‐specific selection pressure on the organisms associated with them; for example, strains of *E. coli* associated with plants are a distinct phenotype and make an independent phylogroup different from that purified from mammalian hosts (Méric, Kemsley, Falush, Saggers, & Lucchini, 2013). *E. coli* strain USML2 has been shown to be a growth‐promoting endophyte in leaves of the oil palm (*Elaeis guineensis*; Tharek, Sim, Khairuddin, Ghazali, & Najimudin, 2017). Different species of *Enterobacter* have been reported as rhizobacteria associated with different plant species (*E. sakazakii* and *E. agglomerans* with soybean; *E. cloacae* with citrus, maize, and soybean; and *E. asburiae* with sweet potato). These species possess multiple growth‐promoting traits and enhance plant growth (Ramesh, Sharma, Sharma, Yadav, & Joshi, 2014). Recent studies suggest that *E. coli*and *Enterobacter* also occur as endophytes in different plants and enhance nutrient uptake and growth of their hosts plants and are useful for environmental clean‐up (Santoyoa, Moreno‐Hagelsieb, Orozco‐Mosqueda, & C., & Glick, B.R., 2016). Based on these examples, we propose the use of filamentous phages to enable bacterial inocula to colonize contaminated habitats, control phytopathogens, and promote plant growth for ecorestoration.

Filamentous phage‐infected *P. aeruginosa*shows high potential to colonize different habitats because of its ability to form biofilms, which have a liquid crystalline organizational structure (Rice et al., 2009; Secor et al., 2015). The biofilm is not an inert structure but a cooperative and interactive network that develops into an ecologically cohesive microbial community, which can rapidly colonize the target site (Hengzhuang, Wu, Ciofu, Song, & Høiby, 2011, 2012; Høiby et al., 2011; Høiby, Bjarnsholt, Givskov, Molin, & Ciofu, 2010). In fact, Pf1‐infected *P. aeruginosa*PAO1 strains outcompete the noninfected strains in forming a biofilm and also form a cohesive group for exchanging genes—exchanges from which the noninfected strains are excluded (Whiteley et al., 2001). Such gene exchanges within a biofilm help the bacterial hosts to develop into superinfective phenotypes that can adapt to and colonize new surfaces effectively (Rice et al., 2009; Webb et al., 2004). Lytic phages specific to native bacteria can also create a niche for the bacterial inocula (Kuykendall & Hashem, 1998; van Elsas & van Overbeek, 1993). Therefore, a consortium of lytic phages, filamentous phages, and the target bacterial inocula should be tested for improved colonization by the bacterial inocula of the contaminated site (Figures 3, 4).

Further, the genome of a filamentous phage itself can be manipulated to increase the ability of bacterial hosts that make up the inoculum to colonize diverse environments. Immediate opportunities for such manipulation are available in some plant‐associated bacterial genera such as *Pseudomonas*, *Ralstonia*, and *Xanthomonas* for which filamentous phages have already been reported (Table 5). At the same time, the little‐explored plant growth‐promoting bacteria such as *Acinetobacter*, *Clostridium*, *E. coli*, *Enterobacter*, *Propionibacteria*, *Shewanella*, and *Vibrio*(Table 2) need immediate attention. Concerted efforts are required to extend the similarities in the relationships between filamentous phages and bacteria as revealed in laboratory tests to the bacteria used in commercial formulations and for ecorestoration (Table 1).

### 4.4 Phages to promote community assembly and evolution

A bacterial strain with high genetic stability confers desirable benefits in terms of plant growth and soil health after inoculation; however, successive generations of the strain should also have the ability to diversify and adapt to the changing environment (Sharma, Mishra, Mohmmed, et al., 2011). Filamentous phage may promote genetic stability as well as genetic diversity in a bacterial population depending upon the relative proportions of the filamentous phages and their bacterial hosts.

Environmental stress promotes genetic instability in bacteria; as a result, desirable bacterial phenotypes progressively disappear from the population (Mohmmed, Sharma, Ali, & Babu, 2001; Rau et al., 2009; Sharma et al., 2005; Sharma, Mishra, Mohmmed, et al., 2011; Sharma, Mishra, Rau, & Sharma, 2011). Plasmids, which are extrachromosomal genetic elements, carry environmentally relevant gene(s) in bacteria and help them to adapt to specific niches or confront environmental challenges. Often, the desired bacterial phenotype is lost after inoculation because the plasmid that encodes ecologically competitive gene(s) is lost (Bergstrom, Lipsitch, & Levin, 2000; Hall et al., 2015; Hall, Wood, Harrison, & Brockhurst, 2016; Harrison & Brockhurst, 2012; Mohmmed et al., 2001). At a high phage‐to‐bacterium ratio, infection by the filamentous phage inhibits conjugation in *E. coli*population (K38 and TOP10F) and ensures genetic stability (Boeke et al., 1982; Lin et al., 2011; Table 5). However, the filamentous phage and the *F* ^−^ recipient bacterial cells compete for a common receptor (pilus) of F^+^ donor bacterial cells. Therefore, the ratio of phage to *F* ^−^ recipient bacterial cells determines which of the two events will be more frequent: infection of F^+^ donor bacterial cells by the filamentous phage or conjugation between F^+^ and *F* ^−^ bacterial cells (Novotny, Knight, & Brinton, 1968; Ou, 1973; Wan & Goddard, 2012). The role of filamentous phages in preventing conjugation (Lin et al., 2011; Wan & Goddard, 2012) shows their potential in ecorestoration to maintain genetic stability of bacterial inocula. In fact, the role of protein g3p of a filamentous phage in hindering conjugation process has already been demonstrated. Although a filamentous phage infects and persists within its bacterial host, regular reacquisition by the host may be required to maintain such infected bacteria in a population in sufficient numbers. Also, prior knowledge of the right ratio of filamentous phages to bacterial cells that ensures genetic stability in target bacteria is a prerequisite to using filamentous phages as co‐inoculants.

Independent studies on *E. coli*strains have confirmed the potential of M13 filamentous phage to trigger genetic heterogeneity in an isogenic population of *E. coli*(De Paepe et al., 2010) or to maintain genetic homogeneity in *E. coli* W6 population (Wan & Goddard, 2012; Table 5). Using quantitative analysis, Lin et al. (2011) showed that as the M13 filamentous phage‐to‐*E. coli* ratio increases, the conjugation frequency decreases: A lower ratio favors conjugation and gene exchange, whereas a high ratio lowers the frequency of conjugation. We suggest that strains of bacterial inoculants be examined for the filamentous phages associated with them and the optimum ratio of filamentous phages to their bacterial hosts be determined for triggering gene exchange and genome diversification in the host population.

**Figure 6:**
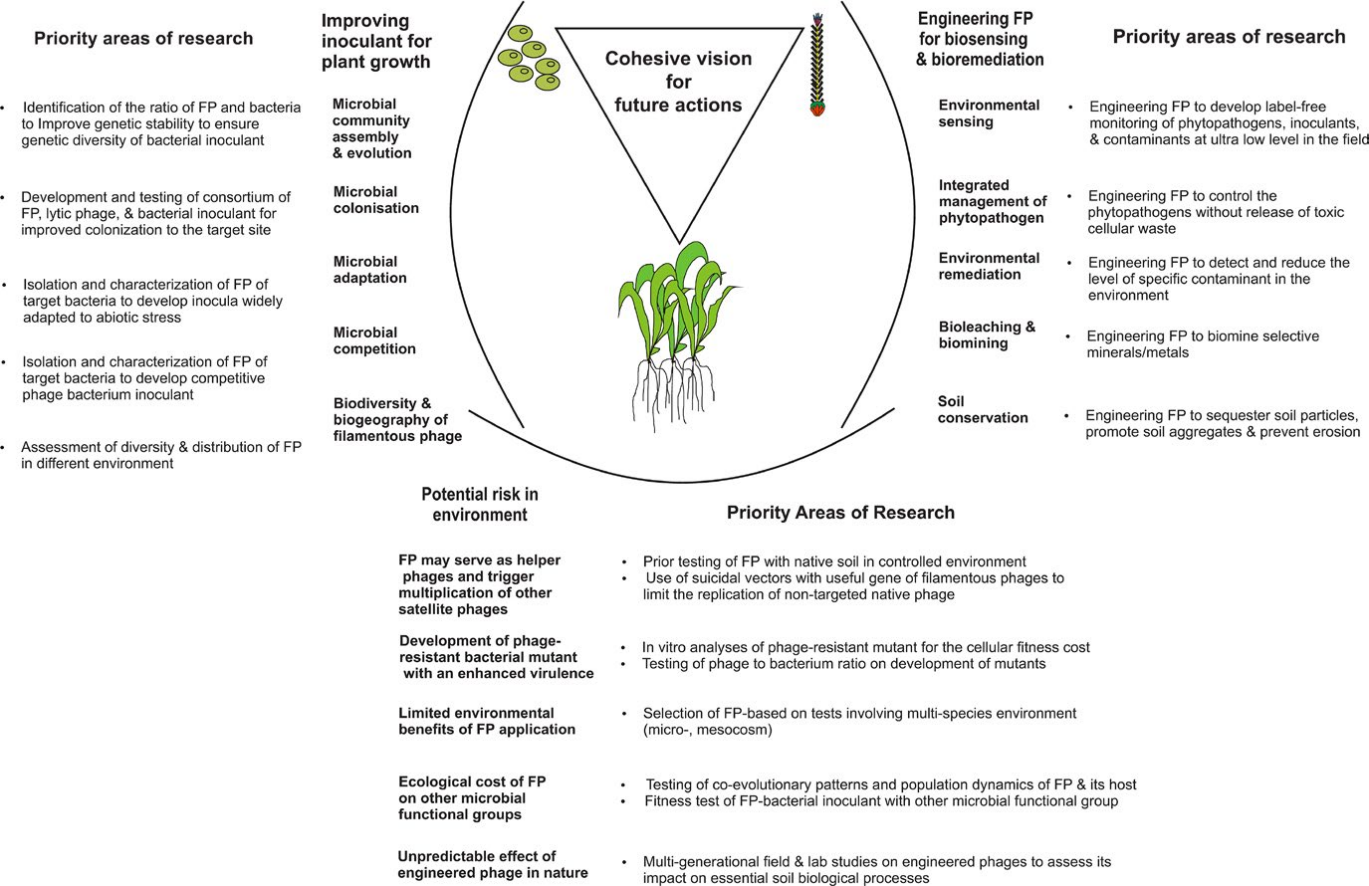
Outline of multi‐branched course of research proposed with a cohesive vision for future actions for environmental application of filamentous phage (i) for improving inoculants for plant growth, (ii) for engineering filamentous phage for biosensing and bioremediation, and (iii) for preventing potential risks of filamentous phage application in the environment. FP, filamentous phage

However, it is important to ask a fundamental question: Are the filamentous phages that trigger genetic stability or promote genetic diversity in bacterial populations different for different bacterial species or is the difference due to the relative proportions of phages and bacteria? To answer this question, we must isolate filamentous phages from different bacterial genera or species from the natural environment and then analyze the impact of their relationships on the ecology of the bacterial hosts using well‐designed laboratory studies. We may use bacterial species with known filamentous phages for identifying the optimal ratio of phages to bacteria for genetic stability and that for genetic diversity in bacterial populations. Such studies will guide in situ genetic engineering of bacterial inocula. Figure 6 outlines a suggested path of research for developing ecologically competitive phage–bacterium inocula based on the ecological impact of the phages‐to‐bacteria ratio.

## 5 ROLE OF FILAMENTOUS PHAGES IN THE MONITORING BACTERIAL INOCULA AND IN DETECTING AND CONTROLLING PLANT PATHOGENS AND CHEMICAL CONTAMINANTS

### 5.1 Phages as sensors

The success of restoration efforts depends on the tools available for detecting and controlling pathogens and contaminants. To evaluate and guide restoration, restoration ecologists monitor inocula, pathogens, and contaminants in time and space (Felici et al., 2008; Harvey, 1993; Lynch et al., 2004; van Elsas, Duarte, Rosado, & Smalla, 1998). However, to monitor multiple contaminants and pathogens in degraded environments, we need different physicochemical and biological methods. Because we employ a consortium of bacterial strains and species to tackle multiple contaminants and pathogens, monitoring a consortium (multiple targets) for survival, colonization, and performance also warrants the use of an array of biological methods.

Compared to the conventional monitoring tools, those based on filamentous phages can be more easily tailored for diverse targets and even for detecting a target when it is present in ultralow levels and that too in real time (Figure 5). Tracking of organisms (bacteria, viruses, etc.) and biological materials (spores, toxins, proteins, and DNA) relies on different methods, which may be (a) microbiological (culture, colony counting, chemical and biological plate assays), (b) biochemical and immunological (enzyme‐ or antibody‐based assays), and (c) biomolecular (marker sequences, gene expression, polymerase chain reaction, etc.). Similarly, the tracking of toxicants involves chemical and analytical methods (titrimetric, spectrophotometric, fluorometric, chemiluminescence, etc.). Despite immense advancements, the use of biochemical and molecular methods for environmental studies continues to face such challenges as high costs, the need to process samples, longer time required for assays, and low sensitivity of methods aimed at detecting biological targets in samples from the environment (Felici et al., 2008; Liu, Li, Khan, & Zhu, 2012). In contrast, filamentous phage‐based sensors are economical, can be used in real time without having to process the samples, and detect targets even when they are present at ultralow concentrations in the environment. Because of these benefits, such biosensors may become an important item in the toolkit of the restoration ecologist.

Filamentous phages offer flexibility in modifying surface proteins to develop target‐specific receptors or probes and ease in combining different transducers or sensor surfaces such as nano‐ or micromechanical, electrochemical, and optical sensing platforms (Bernard & Francis, 2014; Sagona, Grigonyte, MacDonald, & Jaramillo, 2016; Templier, Roux, Roupioz, & Livache, 2016; Tables 6 and 7). Depending upon the sensor platform, biosensors differ in the principles of transduction of the signal and detection of the analyte. The ease and flexibility in tailoring the surface of the filamentous phage using genetic and chemical methods offer a better opportunity to restoration ecologists to develop ultrasensitive, specific, and cost‐effective biosensors for targets that may differ in size and their chemical nature. Such biosensors detect specific targets at ultralow levels in complex environmental samples without any prior treatment. Besides these properties, filamentous phage‐based sensors are stable and robust even under harsh environments and can be reused by regenerating the receptor surface, which makes them ideal for environmental use (Rakonjac et al., 2011; Singh, Poshtiban, & Evoy, 2013). In restoration ecology, such biosensors may serve as bioindicators (specific to bacteria, viruses) or as biomarkers (specific to pathogen‐specific biomolecules such as DNA and protein or to a specific biological activity). Because they are robust, such biosensors may track different biomaterials in complex environmental samples both in vivo (on plant surfaces and inside tissues) and in vitro (Table 5).

**Table 6:** Contributions of modified filamentous phages in detection and control of pathogens in environment

Filamentous phage	Modification in filamentous phage	Biosensor type and working principle	Target biomaterial	Sensitivity/specificity	References
**Phages in theory of environmental sensing:** *As an agent to develop sensitive biosensor to track pathogens*					
E2 from fd and 7b1 from M13 phage libraries	Expression of *Salmonella typhimurium*‐specific peptide (VTPPTQHQ) on pVIII	**Quartz crystal microbalance (QCM):**Modified phage immobilized on QCM platform detects the pathogen based on Piezoelectric effect.	*Salmonella typhimurium*	Detects 10^2^ cells/ml with a response time of <180 s	Olsen et al. (2007)
JRB7, clone from f8/8 landscape phage library	Expression of *B. antracis*‐specific peptide on pVIII	**Magnetoelastic resonators (ME):**Recombinant phage immobilized onto ME platform coated with gold surface binds to antigen	*B. anthracis* spores	Detects 10^3^ spores/ml in vitro conditions	Shen, Lakshmanan, Mathison, Petrenko, and Chin (2009)
E2 derived from fd landscape phage library	Expression of *S. typhimurium‐*specific peptide on pVIII	**Magnetoelastic resonators (ME):**Phage nanoprobe immobilized on ME detects binding of pathogen as decrease in resonance frequency	*S. typhimurium*	Detects 5×10^2^ cfu/ml of pathogen on the surface of tomato	Li, Johnson, et al. (2010), Li, Li, et al. (2010)
M13	M13 displaying peptide specific to target pathogen	**Lithographically patterned nanowire‐electrodeposition (LPNE):**Virus functionalized nanowires developed by grafting modified phage on poly 3,4‐ethylenedioxythiophene (PEDOT) would bind specific antigen.	Specific antigen on targeted pathogen	Detects antigen (20 nM–99 nM) in in vitro and in vivo conditions	Arter, Taggart, Mclntire, Penner, and Weiss (2010)
M13 (Phagemid M13KO7)	Expression of the reporter gene, alkaline phosphatase on pVIII with the help of helper phage M13KO7	**Amperometric Electrochemical Biosensors:**Phage immobilized on the electrode detects the target organism in environmental samples based on the activity of reporter enzyme	*E. coli*TG1	Detection of 1 cfu/ml in <3 hr; 10‐fold increase in the sensitivity of the biosensor (due to enzyme activity) as compared to other phage‐based biosensors	Neufeld et al. (2005)
M13	Expression of prostate‐specific membrane antigen (PSMA) specific peptide (CALCEF ‐LG) on pVIII	**Electrochemical impedance spectroscopy (EIS):** Electrode developed by covalent binding of phage to the gold surface generates electrical signal on binding of antigen	PSMA	120 nM of the target protein	Yang et al. (2006)
M13	pH‐responsive cyanine dye (HCyC‐646) is covalently attached to pVIII	**Near Infrared Fluorescence Ratiometric pH Imaging**: A bright pH‐responsive ratiometric imaging platform, developed by ligation of dye to the phage surface, measures the emission signal on pH change	Optically diffused tissue	Pathogenesis associated changes in acid–base homeostasis are analyzed by pH measurements both intracellularly and through optically diffused tissue^2008^	Hilderbrand et al. (2008)
Modified M13	Modified pIII binds specifically to target antigens, whereas modified pVIII reacts with signal‐producing gold nanoparticles (Au NPs)	**Single bioanalytical platform for antigen detection and identification:**DNA‐conjugated phage detects (optically/spectroscopically) and identifies the antigen (DNA microarray)	Protein detection and identification	Real time, reagent free detection of antigen (detection limit 25 fmole) and its identification in a high‐throughput manner	Lee, Domaille, and Cha (2012)
R5C2	Expression of streptavidin‐binding peptide NH_2_–ANRLP CHPQFPCTSHE on pVIII	**Opto‐fluidic ring resonator (OFRR) – Lab‐on‐Chip device:**Phage integrated on OFRR platform acts as a receptor for detection of analyte and enables label‐free detection of the analyte in small volume	Protein/DNA/Virus	Real‐time detection of protein/DNA (100 pM; Kd_apparent_ 25 pM) and virus particles (2.3 × 10^3^ pfu/ml)	Zhu et al. (2008)
A clone from f8/8 landscape phage library	Expression of EPRLSPHS peptide on pVIII protein	Modified phage displaying unique landscape on its body binds antigen with extremely high efficiency and specificity	Spores of *Bacillus anthracis*	Up to 70‐fold high specificity to spores of *B. anthracis* than other *Bacillus*spp.	Brigati et al. (2004)
**Phages in theory of antagonism and biocontrol:** *As an agent to biocontrol of phytopathogens*					
M13	Phages encode restriction endonuclease BglII gene (M13R) or λS holin gene (M13S105, M13VIIIS105)	**Genetically modified (GM) phage as bactericidal agents:** Modified phage kills bacterial host without lysis and release of toxin in environment	*E. coli* strain MC4100F¢	>99% killing of pathogen within 2 hr	Hagens and Bläsi (2003)
Pf3	Phage export protein gene is replaced with Bgl*II* endonuclease gene	**Nonreplicating GM phage as bactericidal agents:**Modified phage kills host bacteria without lysis and release of endotoxins	*Pseudomonas aeruginosa*	Phage treatment effectively control *P. aeruginosa* population in in vivo condition with a minimal lethal dose	Hagens, Habel, von Ahsen, von Gabain, and Blasi (2004)
Nonlytic, M13 phagemid	Encode the addiction toxins modules (Gef and ChpBK)	**Recombinant Phage as a Lethal‐Agent Delivery Vehicle:**Modified phage delivers addiction toxins module to elicit programmed cell death in bacterial host	*E. coli* ER2738	94%–98% reduction in in vivo condition within 5 hr	Westwater et al. (2003)
M13mp18 phage	Expression of genes (csrA and ompF) that target multiple antibiotics simultaneously	**Adjuvant therapy to treat antibiotic‐resistant bacteria:**Modified phages trigger SOS and non‐SOS gene networks that are not directly targeted by antibiotics (e.g., aminoglycosides and β‐lactams) kills antibiotic‐resistant bacteria, biofilm cells, and persister cells	*E. coli* EMG2 and RFS289	Modified phage kills antibiotic‐resistant bacteria, biofilm cells, and persister cells	Lu and Collins (2009)
M13	Predominant expression of three glutamic acid (E3) residues on pVIII	**Silverized Antimicrobial Phage Fibers**: Modified phages immobilized to the fibers electrostatically bind silver ions which on subsequent reduction to metallic silver provide antibacterial properties to the fibers	*Staphylococcus epidermidis* and *E. coli*	Biocidal fibers show bactericidal activity up to 300 □m distance within a 2 hr exposure; kills 10^9^ cfu/ml with 20 hr continued exposure	Mao, Belcher, and Van Vliet (2010)
fRSM (φRSM1 or φRSM3)	Phages found in natural association with host bacteria *R. solanacearum* MAFF 106603 and MAFF106611	**Phage therapy reduces virulence of phytopathogen:** Phage‐infected host cells are characterized with reduction in: (i) twitching motility; (ii) number of type IV pili (Tfp); (iii) β‐1,4‐endoglucanase (Egl) activity; (iv) extracellular polysaccharide (EPS) production, and (iii) expression of certain genes (egl, pehC, phcA, phcB, pilT, and hrpB).	*R. solanacearum* MAFF 106603 and MAFF 106611	Loss of virulence to cause wilting to tomato plant (10^5^ cfu wild type trigger wilting within 3 days and cause plant death within 5–7 days)	Addy et al. (2012a)
φXacF1	Phage found in natural association with host bacteria *X. axonopodis* pv. *citri* MAFF673010 and MAFF301080	**Phage therapy reduces virulence of pathogen:**φXacF1‐infected *X. axonopodis* pv. *citri* strains are characterized with lower levels of extracellular polysaccharide production, reduced twitching motility, slower growth rate, and a dramatic reduction in virulence.	*X. axonopodis* pv. *citri* MAFF673010 and MAFF301080	Extreme reduction in virulence, failed to cause citrus canker in lemon even after 4 weeks of post‐infection	Ahmad et al. (2014)

**Table 7:** Contributions of modified filamentous to detect and detoxify the contaminants in environment

Filamentous phage	Modification in filamentous phage	Integrating component and working principle	Target biomaterial/potential target	Sensitivity/specificity	References
**Phages in theories of environmental sensing and remediation:** *As an agent to detect and sequester the contaminants*					
Affinity selected M13 clones	Expression of TNT/TNB‐specific peptide on pIII, minor coat protein; Cy5 dye covalently linked to free amino groups on phage surface	**Phage‐based immunofluorescence:**Modified phage integrated into continuous flow immuno sensor platform detects TNT in environmental samples; fluorescence intensity is taken as a measure of TNT bound to phages	2,4,6‐trinitrotoluene (TNT) and 2,4,6‐trinitrobenzene (TNB) derivatives	ELISA‐based detection of 10 ng/ml TNT in environmentally relevant, nonphysiological medium, artificial seawater	Goldman et al. (2002)
M13 selected from random heptapeptide library	Expression of KPLLMGS, QPKGPKQ and TPTTYKV peptide on pIII	**Mineral Processing:**Modified phages with KPLLMGS and QPKGPKQ bound specifically to sphalerite and those with TPTTYKV showed specificity to both sphalerite and chalcopyrite;	Sphalerite (ZnS) and chalcopyrite (CuFeS2) in environmental sample	Mineral detection within 1 h of treatment at room temperature	Curtis et al. (2009)
VP12 and VP14 selected from P8/8 library	Expression of DSQKTNPS (VP12) and DGQKASNS (VP14) at pVIII	**Mineral aggregation:**Phage with charged and polar residues on the surface induce aggregation and removal of physical contaminants (via salt bridges) from oil sand tailings in a wide range of pH and ionic strength.	Sphalerite (ZnS) and chalcopyrite (CuFeS2)	5 × 10^11^–5 × 10^12^phages/ml induced aggregation of fine clay (<45 μm); 1,012 phage/ml settled ~99% of the mineral ore in 10 s;	Curtis et al. (2011), Curtis et al. (2013)
ScFv phage display library of M13	Phage expressed single chain antibodies (fused heavy and light chain variable domains) on pIII	**Recombinant antibody labeled phage:** Phage with TNT specific fluorescence labeled antibodies detects TNT in environment	TNT and derivatives	Detects TNT in ultralow level (1 ng/ml) in aqueous solution	Goldman et al. (2003)
M13 from ScFv phage display library	Phage expressed single chain antibodies on the coat proteins are selected	**Noncompetitive Immunoassay for Small molecules:**phage expressing fluorescence labeled recombinant antibodies detects morphine in environment by measuring FRET signal	Small analytes like morphine	Detects ultralow levels (5 ng/ml) of morphine within 2 min even in the presence of structurally similar analytes	Pulli, Höyhtyä, Söderlund, and Takkinen (2005)
1G40	Phage expressed reporter gene ( β‐galactosidase) at pVIII	**Surface Plasmon Resonance (SPR) Sensors:** Phage immobilized on the gold surface of SPR biosensor chip detects the substrate based on the activity of reporter enzyme.	Substrate for the reporter gene (e.g., β‐galactose for β‐galactosidase)	Detects contaminant even at 10^−7^M	Nanduri, Bhunia, Tu, Paoli, and Brewster (2007 )
Modified M13mp18	A short random 20 bp DNA sequence is inserted at *Sin*I site of the phage genome	**Bacteriophage biotracing:** Modified phage used for tracing the pollution source in environmental condition; analyses is based on plaque assay and identification of unique sequence	Modified bacteriophage	Detects even 1pfu/ml in presence of wild type (M13) and other closely related phages (F1, Fd); simultaneous tracing of a number of pollution sources is feasible	Daniell, Davy, and Smith (2000)

Micromechanical biosensors have exploited modified filamentous phages to develop sensors for detecting bacterial cells (analyte) at ultralow levels. Quartz crystal microbalances (QCMs), magnetoelastic (ME) resonators, and nano‐cantilevers represent the micromechanical type of biosensors. The analyte mass accumulates on the surface of the micromechanical biosensor and results in a corresponding shift in the vibrational resonance of the transducer. A QCM sensor comprises a piezoelectric plate coated with metallic electrodes on both sides. The modified filamentous phage immobilized on the surface of a QCM detects the wet mass of the target bacteria even in nanograms. Bacterial cells bound to the phage‐displayed probe cause a resonance shift under a magnetic field, which makes detection possible in real time. An ME sensor consists of an amorphous ferromagnetic ribbon, which is an advantage in environmental monitoring due to its small size, low cost, and passive and wireless nature. Detecting a target requires neither prior sample preparation nor enrichment of bacterial cells. Biosensors in the form of QCM with E2 phage modified to express specific peptides detected extremely low numbers of the target pathogen (*Salmonella typhimurium*: 102 cfu/ml) within 3 min (Huang et al., 2007; Lee, Song, Hwang, & Lee, 2013); an ME‐filamentous phage biosensor also detected bacterial pathogen in ultralow numbers (*S. typhimurium*: 50 cfu/ml) on the surface of tomato (Li, Johnson, et al., 2010; Li, Li, et al., 2010); and another ME biosensor with modified JRB7 detected spores of *B. anthracis*(10^3^ spores/ml) in vitro.

Electrochemical biosensors have used filamentous phages to detect chemical changes in a cellular environment: The sensor phages display target‐specific peptides, which detect and report the analyte as a change in current (amperometric sensor), impedance (impedance sensor), and voltage potential (light‐addressable potentiometric sensor). Modified M13 helper phage expresses an electrochemically active reporter, such as alkaline phosphatase, at the surface; the reporter measures the current flow (oxidation‐reduction reaction) and detects the signal in an amperometric sensing system. An amperometric electrochemical biosensor with modified M13 detected *E. coli* TG1 at concentrations as low as 1 cfu/ml by monitoring the activity of the reporter enzyme (Neufeld, Mittelman, Buchner, & Rishpon, 2005). A filamentous phage‐based imaging system also detects pathogen‐related chemical changes (acid–base homeostasis) in optically diffuse tissue (Hilderbrand, Kelly, Niedre, & Weissleder, 2008); for example, phage M13 was modified to ligate a pH‐responsive cyanine dye (HCyc‐646) to pVIII and thus developed into a ratiometric probe. The engineered phage‐based sensor can also use impedance spectroscopy, an electrochemical technique, for such applications. These methods are highly sensitive and easy: For example, a biosensor with engineered M13 filamentous phage covalently attached to a gold electrode measures electrical impedance over a wide range of frequencies (in kHz) and can detect ~120 nanomolar prostate‐specific membrane antigen at signal‐to‐noise ratios greater than 10. Filamentous phage‐based light‐addressable potentiometric sensors (LAPS) represent another such biosensor, which is composed of a semiconductor‐insulator base activated by directed light pulses. These label‐free biosensors detect target enzyme activity or cellular pH, redox condition, or ion gradients, and LAPS have proved flexible enough to modify covalently with as many as four different phages.

Optical sensors, which rely on either spectrometry‐based or resonance‐based sensing, have the potential to detect target biomolecules at ultralow levels. Spectrometry‐based methods such as UV/Vis spectrometry, bio/chemiluminescence, fluorescence or phosphorescence spectrometry, and infrared spectrometry measure changes in intensity at a particular wavelength whereas resonance‐based methods such as surface plasmon resonance (SPR), fluorescence resonance energy transfer (FRET), and colorimetry measure changes in chemical properties upon a change in the wavelength. Opto‐fluidic ring resonator (OFRR) integrates microfluidics and photonic sensing technologies to develop an ultrasensing platform for detecting the target at ultralow levels (as low as nanoliters or at concentrations of 10 pg/mm^2^). Low cost, high sensitivity, and good reusability of filamentous phage‐based OFRR biosensors make them a promising platform for detection of biomolecules in the environment (Table 6): An OFRR (a lab‐on‐a‐chip device) comprising immobilized phage R5C2 on silicon microfluidics detected targeted protein/DNA at picomolar levels in real time (Suter et al., 2008; Zhu, White, Suter, & Fan, 2008; Zhu, White, Suter, Zourob, & Fan, 2008).

Filamentous phage‐based field effect transistors (FET) can potentially track target viruses in environmental samples: FET comprise a source, a gate, and drain electrodes and detect the target biomolecule in a fluid system based on the change in current–voltage characteristics of the transistor (electrical transduction). Such an electrical biosensor houses a modified filamentous phage as a probe and a semiconductor device as a transducer. The miniaturization and integration into a small chip make the biosensor useful for high‐throughput analysis, whereas integration of nanowires provides an opportunity to develop next‐generation ultrasensitive biosensors: label‐free polymer‐nanowires‐based FET transistors or chemiresistors as biosensors detected bacterial pathogens even at 1 cfu/ml and phages at 10^3^ pfu/ml in untreated environmental samples (Lee et al., 2013).

Thus, filamentous phages as biosensors are fast, reliable, and easy to use and do not rely on costly probes and are particularly suitable for environmental restoration programs because they are highly specific and highly sensitive, can be mass‐produced cheaply, and can withstand harsh environments.

### 5.2 Phages for biological control

Quick detection and control of phytopathogens facilitate plant recruitment and restoration of vegetation (Al‐Karaki, 2013; Barea, 2015; Bashan, 1998; Sharma et al., 2005). Filamentous phages (modified and natural) can help in detecting and controlling targeted pathogens in environmental samples containing a mixture of even closely related strains. Filamentous phages control the emergence of bacterial pathogens through their effects on the physiology of their bacterial hosts (Table 7; Figure 5). Recombinant filamentous phages have been developed to target such bacterial phytopathogens as *Pseudomonas* (*P. putida*, *P. aeruginosa*), *E. coli*(Hagens & Bläsi, 2003), and *Ralstonia solanacearum* (Yamada, 2013) and filamentous phages have been genetically modified to be antagonistic toward bacterial pathogens through (a) expression of restriction endonucleases, (b) initiation of programmed cell death, (c) development of sensitivity to antimicrobial substances, and (d) development of oxidative burst in host cells (Table 6).

Filamentous phages have been modified as environmentally safe biocontrol agents for target pathogens. Filamentous phages are host‐specific but their nonlytic life cycles initially limited their use in biological control of pathogens (Loc‐Carrillo & Abedon, 2011; Henry et al., 2015; Mai‐Prochnow et al., 2015). However, filamentous phages may serve as a vehicle for delivering toxins into target pathogenic strains—the nonlytic life cycle then becomes an advantage because it prevents the release of toxic cellular waste or endotoxins into the immediate environment (Goodridge, 2010; Lu & Koeris, 2011; Viertel, Ritter, & Horz, 2014) and also prevents undesirable changes in the structure and functioning of the microbial community due to the release of toxic waste from the lysed pathogenic cells. For example, a modified M13 phage delivers “addiction toxin” genes (Gef and ChpBK), which triggers programmed cell death in target bacteria in vivo (Table 6). Such “suicide” systems have shown their potential to control some environmentally significant bacteria, namely *Pseudomonas* (*P. putida*, *P. aeruginosa*) and *E. coli*(Hagens & Bläsi, 2003). Phage M13 has been modified to encode restriction endonuclease (*Bgl*II) for killing the target bacteria with efficiency comparable to that of lytic phages. Therefore, modified filamentous phages not only control the target bacteria but also minimize the risk of the toxins being released into soil. Considering these benefits, we believe that filamentous phage‐based biocontrol of pathogens will also add to the efficacy of bacterial inocula in restoration of vegetation.

The use of filamentous phages to control pathogens that are resistant to multiple antibiotics is yet to win the attention of restoration ecologists that it deserves. For example, M13mp18 modified phage targets SOS and non‐SOS networks of bacterial hosts and controls antibiotic‐resistant and persister strains of *E. coli*(EMG2, RFS289; Lu & Collins, 2009). Also, silverized antimicrobial phage fibers developed with E3 modified M13 kill bacterial contaminants in water during filtration through phage‐coated fibers (Mao et al., 2010; Table 6). Fusion phages (fd‐pIIICTX) can control diverse pathogens as effectively as phage cocktails or mixtures can (Pires, Cleto, Sillankorva, Azeredo, & Lu, 2016). In some cases, natural filamentous phages can also reduce the pathogenicity of their bacterial host. Such filamentous phages offer an opportunity for environmentally safe biocontrol of pathogens. For example, Addy et al. (2012a) reported a filamentous phage (φRSM) of the wilt‐causing bacterial pathogen *Ralstonia solanacearum*: φRSM infection made the pathogen less virulent and thus prevented damage to its host plants (Yamada, 2013). Ecologists have also been interested in φRSM because *R. solanacearum*is a serious threat to phytorestoration programs (Zhang et al., 2017), for example to programs to grow tobacco for ecorestoration of nutrient‐deficient and contaminant‐rich soils. In fact, degraded soils show greater abundance of not only *R. solanacearum*but also of pathogenic members of *Pseudomonas*, *Erwinia*, and *Xanthomonas*. Controlling *R. solanacearum* is a challenge because it survives as a latent infection in indigenous weeds for many years (Hayward, 1991; Wenneker et al., 1999). However, it would also be useful to isolate and engineer filamentous phages of other pathogenic bacteria and test the potential of these phages to control the pathogens of other wild plants. Such use of filamentous phages to lower pathogenicity, virulence, and spread of dreadful phytopathogens may mark a new milestone in restoration programs.

The increasing numbers of bacterial genera discovered to have an association with filamentous phages and the ease with which the phage genome can be modified to produce antimicrobial peptides represent an untapped but most promising opportunity for biocontrol of pathogens. Using suitably modified filamentous phages along with other biocontrol agents can revolutionize the integrated management of phytopathogens. However, these phages are at times unstable in their host populations, even when they do not result in the evolution of host resistance (Lerner & Model, 1981). This characteristic, and the loss of filamentous phages during molecular applications (Mai‐Prochnow et al., 20155), is obstacles to their use as inhibitors of pathogenic bacteria.

However, other species or strains of *Ralstonia*also develop symbiotic endophytic associations with plant species that show high potential for phytoremediation. For example, poplars (*Populus* spp.) and willows (*Salix* spp.), which are used for phytoremediation, also carry *Ralstonia*as an endophyte besides *Acinetobacter*, *Pseudomonas*, and *Xanthomonas*. *Ralstonia*is also an endophyte of *Anthurium*, *Brassica juncia*, *Geranium*, *Gerbera*, *Heliconia*, *Mimosa*, *Salvia*, sunflower, tobacco, *Verbena*, and *Zinnia*, which are also used for phytoremediation of sites contaminated with inorganic and organic pollutants (Ikeura et al., 2016; Kabra, Khandare, Kurade, & Govindwar, 2011; Liu, Xin, & Zhou, 2017; Madera‐Parra, Peña‐Salamanca, Pena, Rousseau, & Lens, 2015; Mahdieh, Yazdani, & Mahdieh, 2013). For example, *R. eutropha* was effective in combination with sunflower (*Helianthus*) for phytoremediation of sites contaminated with Cd and Zn (Marques, Moreira, Franco, Rangel, & Castro, 2013), with maize for phytoremediation of sites contaminated with Cd (Moreira, Marques, Franco, Rangel, & Castro, 2014), and with *Juncus acutus* for biotransformation of Cr(VI) (Dimitroula et al., 2015). Filamentous phages that affect members of *Ralstonia*are useful in controlling phytopathogenic strains of the bacteria and in improving the efficacy of endophytic bacterial strains used in phytoremediation.

### 5.3 Phages for remediation of contaminated sites

Restoration ecologists use microbial technologies for remediation of contaminated sites and for restoring ecosystem goods and services (UNCCD, 2013), and filamentous phages have been engineered to detect contaminants (physical, organic, and inorganic) and to remove or to reduce them in the environment. To assess the success of such efforts, it is necessary to examine the environment for the presence of such contaminants (Brown, 1997; Henry et al., 2015; Viertel et al., 2014; Table 3).

Phages display specific peptides on phage coat proteins (pVIII and pIII), which have been used for detecting, binding to, or decomposing the contaminants (Henry et al., 2015; Nambudripad, Stark, & Makowski, 1991; Petrenko & Makowski, 1993; Thiriot, Nevzorova, & Opella, 2005). Display technology can screen billions of potential toxicants and help in developing fusion phages that retain not only their infectivity and immunogenicity but also their degradation ability (Lerner, Benkovic, & Schultz, 1991). Such properties of modified phages make them potential co‐inoculants with the host bacteria to extract specific pollutants from the environment. For remediation of contaminated soils, phage inoculation thus represents a cost‐effective method of setting up a factory in situ to produce large quantities of contaminant‐specific biomolecules for remediation of the soil environment. This environment‐friendly approach may not be a permanent solution for bioremediation but it is worthwhile to test its potential to reduce the concentration of contaminants in the environment.

Modified filamentous phages also offer another method of separating and processing minerals (bioleaching or biomining) for restoring abandoned mines. For example, modified phages show specificity and selectivity in binding to environmental sources of sphalerite (ZnS, a major ore of zinc) and chalcopyrite (CuFeS_2,_a major ore of copper; Table 7). These phages separate sphalerite efficiently despite the presence of such natural contaminants as silica (a waste mineral) and pyrite (Curtis et al., 2009). The modified phages can potentially mine minerals and metals selectively from natural ore and remediate abandoned mines by acting on waste and industrial scrap and thus contribute to economic and ecological security.

Filamentous phages VP12 and VP14 have been modified to display a specific peptide (DSQKTNPS on pVIII) that sequesters physical pollutants (fine silt and clay particles; Curtis et al., 2011; Curtis et al., 2013). In fact, the mechanism by which the peptide binds to chalcopyrite has also been elucidated: The two phages sequester on their surface particles smaller than 45 µm in diameter under a wide range of pH (3–11) and cation concentrations. The potential of such phages to aggregate soil particles and thereby prevent erosion can be tapped as a novel strategy in soil management.

Phages have also been modified to display peptides (12‐mer) specific to organic contaminants, namely 2,4,6‐trinitrotoluene (TNT) and 2,4,6‐trinitrobenzene (TNB; Table 7). Such modified phages can detect the target organic contaminants at ultralow levels (10 ng/ml) even in heterogeneous environmental samples (Goldman et al., 2002). Modified M13 phages displaying antibodies detected TNT and its derivatives even at concentrations of 1 ng/ml (Goldman et al., 2003). The modified phages displaying specific antibodies are highly selective and sensitive and can detect even minute quantities of such contaminants as morphine at ultralow levels (5 ng/ml within 2 min) in environmental samples (Pulli et al., 2005). Therefore, co‐inoculation of modified phages with plant growth‐promoting bacteria will serve as a supplementary biotechnology to sequester and detoxify targeted contaminants and to speed up revegetation of degraded lands.

## 6 PRIORITY AREAS OF RESEARCH TO MINIMIZE THE POTENTIAL RISK TO THE ENVIRONMENT FROM THE APPLICATION OF FILAMENTOUS PHAGES

So far, filamentous phages have been presented in a positive light. However, as with invasive species and transgenic organisms, the potential risks from introducing filamentous phages that are foreign to degraded soils must not be overlooked. Even if a phage is native to the soil, phage inoculation may disturb the phage‐to‐bacteria ratio, which is crucial to many biological processes in soil (Meaden & Koskella, 2013; Reyes et al., 2010). Because bacterial inoculation aims to restore adversely affected soil processes in degraded lands, the risk from using modified or natural filamentous phages is minimal (Sharma et al., 2015; Sharma, Mishra, Mohmmed, & Babu, 2008; Sharma, Mishra, Rau, et al., 2011). Based on a cost‐benefit analysis, we maintain that the benefits of filamentous phages outweigh the risks from deploying them in damaged ecosystems (Figure 6). Also, the impact of inoculation with filamentous phages is hard to predict unless we have prior knowledge of their host range and the proportion of filamentous phage‐infected bacteria in bacterial populations. Based on the low density and diversity of bacterial communities in degraded environments, we believe that the immediate benefits of phages outweigh the possible risks (Figures 5and 6).

Expansion of the host range of nonnative or modified filamentous phages and the activation of unknown silent phages in bacterial populations in inoculated soils are other potential risks from phage application. Filamentous phages are highly host‐specific and rarely extend their host range to include other strains, species, or genera (Hyman & Abedon, 2010; Piekarowicz et al., 2014). To preclude the possible risk of filamentous phages serving as helper phages for the multiplication of satellite phages (Rakonjac et al., 2011), it is essential to conduct prior experiments with native soils (Figure 6). Alternatively, to limit the replication of nontargeted phages in soil, we may load the useful genes from filamentous phages into a suicide vector (Addy, Askora, Kawasaki, Fujie, & Yamada, 2014; Huber & Waldor, 2002; Martínez & Campos‐Gómez, 2016; Pant et al., 2015). To ascertain the possible impact of introduced phages on the functioning of microbial communities, we need to generate relevant knowledge using ecologically relevant laboratory‐ and field‐based studies in a multi‐species environment (microcosm or mesocosm).

Evolution of phage‐resistant but virulent bacterial mutants is another common concern (Hosseinidoust, Ven, & Tufenkji, 2013). However, in the environment, selection may work against the multiplication of such mutants. Bacteria with mutated pili develop resistance to filamentous phages but show reduced fitness. It may be noted that the pilus helps a bacterium to adhere to suitable objects, move, colonize, and spread into the environment. At the same time, the phages‐to‐bacteria ratio affects the frequency of conjugation in bacterial populations. Therefore, we propose that the population dynamics of filamentous phages and their bacterial hosts in a given environment be examined before deploying filamentous phages for remediation and the impact of population dynamics on the frequency of conjugation and on the functioning of host bacteria be estimated to develop strategies for the use of filamentous phages.

Finally, we need to consider the costs and benefits of the environmental use of filamentous phages based on in vitro studies, which are characterized by a stable environment with few limiting factors. It is possible that the benefits will be lower in the fluctuating natural environments with many limiting factors. However, even marginal improvements in the efficacy of bacterial inoculants due to filamentous phages are worthwhile and particularly important in restoring degraded lands. The costs and benefits are context dependent. The effect of a phage on the bacterial cell is likely to have bottom‐up consequences on bacterial populations, on microbial communities, and on other associated organisms (plants and animals). Such effects of phage inoculation on interactions between organisms will have ecological costs in terms of its impacts on other functional groups of microbes such as associative cooperators, competitors, and predators. Therefore, we suggest that the ecological cost of the application of filamentous phages be estimated and used as the foundation for developing suitable methods for co‐inoculation with bacteria and filamentous phages (Figure 6).

## 7 CONCLUSIONS

Theories related to the ecology of soil microbes have guided microbe‐assisted environmental restoration programs. Applying such theories to phage–bacterium interactions will improve microbial inoculation technologies for revegetation of degraded lands. Growing knowledge of the ecology of filamentous phages of different bacterial genera in diverse environmental settings shows the potential of phages as an emerging bioresource suitable for environmental applications. Co‐inoculation of bacteria with filamentous phages will increase the ecological and evolutionary potential of microbial communities in degraded lands because filamentous phages will help bacterial inocula to colonize degraded sites subjected to multiple abiotic and biotic sources of stress. Further, the ease with which the genome of filamentous phages can be manipulated to express a range of peptides and proteins makes such phages a real‐time sensing tool for environmental restoration. Sensors based on filamentous phages can detect inocula, pathogens, and contaminants in environmental samples at ultralow levels and will not only contribute to more informed decisions related to restoration but also save time and resources. We recommend that restoration ecologists exploit filamentous phages to enhance the ecophysiological capabilities of host bacteria and to find new filamentous phages and understand their ecological relevance. Researchers in environmental remediation will benefit from studying the ecology of filamentous phages to shape bacterial populations for a given purpose; in turn, the exercise of shaping bacterial populations will help the researchers to understand the ecology of phage better. The priority research areas identified in this review will help to realize the potential of phage–bacterium co‐inoculation in environmental restoration and, at the same time, minimize the possible risks from deploying phages. Such use of filamentous phages can usher a tectonic shift in the science and practice of ecorestoration.

## CONFLICT OF INTEREST

Authors declare no conflict of interest.

## AUTHOR'S CONTRIBUTION

Conceived the hypothesis: RSS, VM, SK. Analyzed the literature/data: SK, VM, RSS, PK. Wrote the paper: RSS, SK, VM, PK.

## Data Availability

All the data used for developing the proposals and recommendations presented in this paper were sourced from published studies, and appropriate references are provided.
